# CR1(+) tumor-associated macrophages orchestrate an immunosuppressive niche in hepatocellular carcinoma: a genetic and multi-omics dissection

**DOI:** 10.1186/s12967-026-08301-z

**Published:** 2026-05-25

**Authors:** Zhengjian Wang, Zhe Wang, Xuda Ji, Liping Zhao, Kai Zheng, Wen Yu, Hanzhe Zhang, Hong Chang, Fangfeng Liu

**Affiliations:** 1 Department of Hepatobiliary Surgery, Shandong Provincial Hospital Affiliated to Shandong First Medical University, Jinan, Shandong 250021 China; 2 Shandong Provincial Hospital, Shandong University, Jinan, Shandong 250021 China; 3 Key Laboratory of Engineering of Shandong Province, Shandong Provincial Hospital, Jinan, Shandong 250021 China; 4 Medical Science and Technology Innovation Center, Shandong First Medical University & Shandong Academy of Medical Sciences, Shandong, 250021 China; 5 Department of Hepatobiliary and Pancreatic Surgery, The Third People’s Hospital of Yunnan Province, Yunnan, 650000 China

**Keywords:** Complement receptor 1 (CR1), Hepatocellular carcinoma, Tumor-associated macrophages, M2 polarization, CD8^+^ T-cell exhaustion, Mendelian randomization, Tumor microbiome

## Abstract

**Background:**

Hepatocellular carcinoma (HCC) remains a major global health burden and a leading cause of cancer-related mortality. Advanced disease is characterized by a profoundly immunosuppressive tumor microenvironment (TME) and limited durable responses to therapy. However, the upstream genetic determinants that drive tumor-associated macrophage (TAM) dysfunction in HCC remain poorly defined. Using an integrative genetic and multi-omics framework, we investigated complement receptor 1 (CR1) as a candidate regulator of this immunosuppressive niche.

**Methods:**

We combined Mendelian randomization (MR) and metabolite mediation analyses with bulk, single-cell, and spatial transcriptomics to define the role of CR1 in HCC. Public datasets included the TCGA-HCC cohort, a single-cell RNA-sequencing dataset comprising 53,474 high-quality cells from 21 samples, and two spatially profiled HCC sections. Clinical validation was performed in 30 paired HCC and adjacent liver tissues. Functional assays were conducted in THP-1-derived macrophages using CR1 gain- and loss-of-function approaches, phagocytosis assays, and macrophage-CD8^+^ T-cell co-culture experiments.

**Results:**

MR analyses implicated CR1 in HCC susceptibility at both the protein and transcript levels. pQTL analysis linked genetically predicted circulating CR1 levels to HCC risk (IVW OR = 1.403, *p* = 0.017), and mediation analysis identified specific metabolites as candidate intermediates. Integrative multi-omics analyses showed that CR1 was preferentially enriched in TAMs, spatially co-localized with the M2 marker CD206, and associated with reduced CD8^+^ T-cell infiltration, enhanced T-cell exhaustion signatures, advanced clinicopathological features, and poorer survival. In 30 paired clinical samples, CR1-high tumors exhibited increased M2-like macrophage accumulation and reduced CD8^+^ T-cell infiltration. Functionally, CR1 overexpression drove macrophages toward an M2-like phenotype, enhanced phagocytic activity, increased PD-L1 expression, and suppressed CD8^+^ T-cell proliferation as well as IFN-gamma and granzyme B production, whereas CR1 knockdown produced the opposite phenotype.

**Conclusions:**

Our study provides the first integrated genetic, spatial, and functional evidence that CR1^+^ TAMs constitute a clinically relevant immunoregulatory axis in HCC. These findings extend current understanding of complement-associated immunosuppression beyond canonical complement cascade activity and support CR1 as a candidate biomarker and therapeutic target for macrophage reprogramming, with potential translational relevance for combination strategies involving immune checkpoint blockade.

**Supplementary information:**

The online version contains supplementary material available at 10.1186/s12967-026-08301-z.

## Graphical Abstract

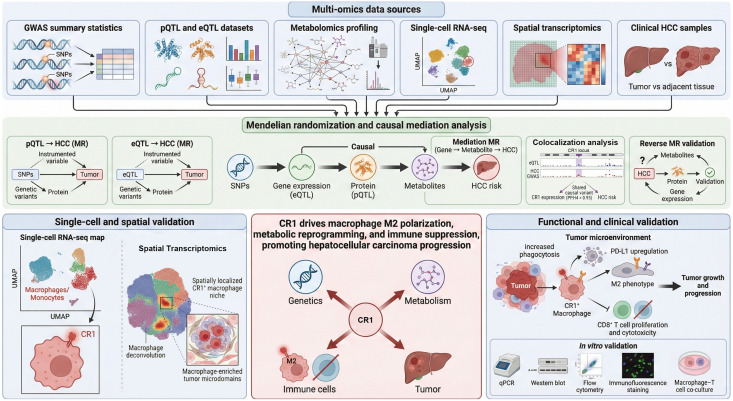

## Intrnoductio

Hepatocellular carcinoma (HCC) is among the most lethal malignancies worldwide and remains a major contributor to global cancer-related mortality, particularly in populations burdened by chronic hepatitis virus infection, cirrhosis, and the growing epidemic of metabolic dysfunction-associated liver disease [1–5]. Although surveillance, surgical resection, ablation, locoregional therapy, targeted therapy, and immunotherapy have improved the management of selected patients, the overall prognosis of advanced HCC remains unsatisfactory [2, 6–9]. This limited therapeutic durability reflects, at least in part, the profoundly immunosuppressive tumor microenvironment (TME) of HCC, which comprises malignant cells, immune populations, stromal elements, metabolic cues, and non-cellular mediators that collectively constrain effective antitumor immunity [10–12].

Before the advent of contemporary targeted agents and immune checkpoint inhibitors, conventional cytotoxic chemotherapy was among the systemic treatment strategies explored for advanced malignancies, including unresectable HCC [13, 14]. In general, chemotherapeutic agents exert antitumor effects by damaging or binding DNA, interfering with mitotic progression, and disrupting biosynthetic processes required for rapid tumor-cell proliferation, and these principles continue to inform anticancer drug development and drug-delivery optimization [15, 16]. However, the role of chemotherapy in HCC has historically been limited by intrinsic chemoresistance, marked tumor heterogeneity, and the frequent coexistence of cirrhosis or impaired hepatic reserve, all of which narrow the therapeutic window and contribute to the modest durability of conventional systemic treatment in this disease [17]. This clinical context further underscores the importance of dissecting the immunosuppressive TME, particularly myeloid-driven mechanisms that may constrain therapeutic efficacy in HCC [18, 19].

Among the immune components of the HCC TME, tumor-associated macrophages (TAMs) and CD8^+^ T cells are particularly important because they jointly determine whether the tumor ecosystem remains immune-permissive or immune-refractory [20, 21]. In many HCCs, infiltrating macrophages acquire an M2-like state characterized by IL-10 and TGF-beta production, increased expression of inhibitory molecules such as PD-L1, and support for regulatory and exhausted lymphocyte states, ultimately weakening cytotoxic T-cell surveillance [22–25]. However, an important knowledge gap remains: the upstream genetic determinants that connect inherited risk signals to TAM polarization, immunometabolic remodeling, and downstream CD8^+^ T-cell dysfunction in HCC are still poorly defined [26–29].

Complement receptor 1 (CR1) is a multifunctional complement receptor classically involved in immune-complex clearance and regulation of complement activation [30, 31]. Recent studies suggest that complement-associated pathways contribute to tumor progression and immune evasion, yet the role of CR1 itself has not been mechanistically defined in the HCC microenvironment, particularly in relation to macrophage biology [32]. Our preliminary integrative analyses identified CR1 as a compelling candidate because it showed a genetic association with HCC susceptibility, strong myeloid-cell specificity, and marked spatial restriction within tumor tissues, suggesting that CR1 may function as a nodal molecule linking host genetics, immunometabolism, macrophage programming, and T-cell suppression [33, 34].

To address this gap, we designed a study that progressed from causal inference to tissue localization and then to functional validation. Specifically, we integrated MR, metabolite mediation analysis, bulk and single-cell transcriptomics, spatial transcriptomics, clinical tissue validation, and macrophage-T-cell co-culture assays to determine whether CR1 drives immune evasion in HCC. To our knowledge, this is the first study to place CR1 within a coherent genetic-metabolic-immune framework in HCC and to demonstrate that CR1^+^ TAMs represent a spatially defined immunosuppressive compartment associated with aggressive clinicopathological behavior and impaired CD8^+^ T-cell function.

## Materials and methods

### Data sources and preprocessing

#### Exposure, mediator, and outcome datasets

All genetic exposure, mediator, and outcome datasets used in this study were obtained from publicly available resources. Protein quantitative trait loci (pQTL) data were derived from the FinnGen study (Release 10; https://www.finngen.fi/en), based on a SomaScan v4 plasma proteomics genome-wide association study (GWAS) of approximately 830 individuals of Finnish ancestry and covering 7,156 proteins [35]. Expression quantitative trait loci (eQTL) data were obtained from the eQTLGen Consortium (https://www.eqtlgen.org), using the Phase I cross-tissue dataset derived from a large-scale meta-analysis of blood transcriptomes from 31,684 individuals [36]. Summary-level GWAS data for circulating metabolites were retrieved from the Canadian Longitudinal Study on Aging, which included 1,091 metabolites and 309 metabolite ratios measured in 8,299 participants [37].

GWAS summary statistics for HCC were obtained from FinnGen Release 12 under the phenotype code C3_HEPATOCELLU_CARC_EXALLC, including 947 clinically validated HCC cases and 378,749 controls, all of European ancestry.

#### Transcriptomic datasets

Transcriptomic datasets used for functional characterization were obtained from three sources. Bulk RNA-sequencing data for HCC were downloaded from The Cancer Genome Atlas (TCGA) data portal (https://portal.gdc.cancer.gov/). Level 3 HTSeq-fragments per kilobase of transcript per million mapped reads (FPKM)-normalized expression profiles and corresponding clinical information were retrieved, comprising 374 tumor samples and 50 adjacent non-tumorous liver tissues. Single-cell RNA-sequencing (scRNA-seq) data were obtained from the Gene Expression Omnibus (GEO) database (https://www.ncbi.nlm.nih.gov/geo/) under accession number GSE149614, which includes 21 samples (13 tumor tissues and 8 normal liver tissues). Spatial transcriptomics (ST) data were also downloaded from the GEO database under accession number GSE245908 and consisted of two spatially profiled HCC tissue sections.

### MR and mediation analyses

#### Instrumental variable selection and causal estimation

To infer potential causal relationships between exposures and HCC, we applied a two-sample MR framework [38]. For each exposure (pQTLs, eQTLs, and circulating metabolites), single-nucleotide polymorphisms (SNPs) associated at a genome-wide significance threshold of *p* < 1 × 10^-5 were selected as the initial candidate instrumental variables (IVs). Linkage disequilibrium (LD) clumping was then performed using PLINK software with an r^2 threshold of 0.001 and a 10,000-kb window to ensure IV independence. The F-statistic was calculated for each IV, and only strong instruments with F > 10 were retained for subsequent analyses.

The inverse-variance weighted (IVW) method was used as the primary approach for estimating causal effects, complemented by MR-Egger regression, the weighted median method, and the weighted mode method as sensitivity analyses [39]. When an exposure was instrumented by a single SNP, causal estimates were derived using the Wald ratio method.

#### Sensitivity analyses and mediation analyses

To evaluate the robustness of the MR findings, multiple sensitivity analyses were conducted for all significant causal associations. Heterogeneity among IVs was assessed using Cochran’s Q test. Horizontal pleiotropy was evaluated using the MR-Egger intercept test and the MR-PRESSO (Mendelian Randomization Pleiotropy RESidual Sum and Outlier) global test [40]. Leave-one-out analyses were further performed to determine whether the overall causal estimates were disproportionately driven by any single SNP.

For exposure-outcome pairs with significant causal effects identified in the primary MR analyses, we subsequently performed a two-step mediation analysis to investigate the potential mediating role of circulating metabolites. The indirect (mediated) effect was calculated as the product of the path coefficients beta1 (exposure ->mediator) and beta2 (mediator ->outcome), and the proportion mediated was defined as (beta1 × beta2)/beta3, where beta3 represents the total effect of the exposure on the outcome. A metabolite was considered a reliable mediator only if all of the following criteria were satisfied: (i) both the exposure-mediator and mediator-outcome associations reached statistical significance using the IVW method (*p* < 0.05); (ii) colocalization analysis performed with the R package coloc demonstrated a high posterior probability of a shared causal variant (PP.H4 > 0.95) [41]; and (iii) reverse MR analyses provided no evidence supporting a causal effect of the metabolite on the exposure or of the outcome on the metabolite.

### Multi-omics data analyses

#### Drug sensitivity analysis

To evaluate the potential association between key gene expression and chemotherapeutic drug sensitivity, drug-response data were obtained from the Genomics of Drug Sensitivity in Cancer (GDSC) database (https://www.cancerrxgene.org/). Predictive models were constructed using the R package *pRRophetic*, which applies elastic-net regression trained on GDSC cancer cell line transcriptomic profiles and corresponding drug-response data. The trained models were then used to predict the half-maximal inhibitory concentration (IC50) values of chemotherapeutic agents in the TCGA HCC cohort.

Model training was performed using 10-fold cross-validation to ensure robustness and generalizability, and batch effects were corrected using the ComBat algorithm. Differences in predicted IC50 values between the high- and low-expression groups of key genes were compared to assess the impact of gene-expression levels on chemotherapeutic sensitivity.

#### Molecular docking

Three-dimensional protein structures of the key genes were retrieved from the AlphaFold database (https://alphafold.com/) [42]. The chemical structures of candidate compounds were obtained from the PubChem database (https://pubchem.ncbi.nlm.nih.gov/). Molecular docking was performed using AutoDock Vina (v1.2.3), and each docking simulation was repeated nine times [43]. The binding conformation with the lowest predicted binding energy was selected as the optimal model. Docking results were visualized using PyMOL software.

#### Intratumoral microbiome analysis

Intratumoral microbiome profiling was based on previously published microbial abundance data processed using the Kraken algorithm [44]. Three categories of association analyses were performed systematically. First, Spearman correlation analyses were conducted between genus-level microbial abundances and immune cell infiltration scores estimated using the relevant algorithm. Second, correlations between the expression levels of key genes (CR1, YME1L1, and MSI2) and microbial abundances were assessed. Third, expression profiles of previously reported cytotoxic T lymphocyte (CTL)-related gene sets and immunoregulatory molecules were integrated and correlated with microbial abundances to infer the potential impact of microbial composition on antitumor immune activity [45, 46].

#### Single-cell and spatial transcriptomic analyses

scRNA-seq data were processed in the R environment using the Seurat package (v4.3.0) [47]. Rigorous quality control was performed by retaining cells with 200–2,500 detected genes (nFeature_RNA), mitochondrial gene expression < 10%, and total unique molecular identifier (UMI) counts within ±3 median absolute deviations of the median. Potential doublets were identified and removed using DoubletFinder (v2.0.4) [48].

Gene-expression values were normalized using the LogNormalize method with a scale factor of 10,000, and highly variable genes were identified. After regressing out mitochondrial gene content, ribosomal gene content, and cell-cycle effects, batch effects across samples were corrected using the Harmony algorithm [49]. Principal component analysis (PCA) was subsequently performed, and the top 30 principal components were used for Uniform Manifold Approximation and Projection (UMAP) visualization and Louvain clustering. Cell-type annotation was performed by integrating information from the CellMarker and PanglaoDB databases, published literature, and automated annotation using SingleR, and was further confirmed by examining the expression of canonical markers (e.g., EPCAM for epithelial cells, CD68 for macrophages, and CD3D for T cells).

ST data were processed using Seurat (v4.3.0). Raw UMI count matrices were normalized and variance-stabilized using the SCTransform function. PCA and UMAP-based clustering were performed based on the top 3,000 highly variable genes. To deconvolute the cellular composition of each spatial spot, the RCTD (Robust Cell Type Decomposition) algorithm was applied using the annotated scRNA-seq dataset as the reference, enabling estimation of the relative proportions of distinct cell types within each spot [50].

### Experimental validation

#### Clinical sample collection and processing

This study was conducted in strict accordance with ethical guidelines. A total of 30 paired HCC tumor tissues and matched adjacent non-tumorous liver tissues (>2 cm from the tumor margin) were collected from patients undergoing surgical resection. Written informed consent was obtained from all participants **before** surgery. The study protocol was approved by the Ethics Committee of Shandong Provincial Hospital Affiliated to Shandong First Medical University (approval no. SWYX:NO.2025–701).

Immediately after resection, tissue specimens were divided into three portions according to experimental requirements: (i) fresh tissues were processed immediately for flow-cytometric analysis; (ii) one portion of the tissues was snap-frozen in liquid nitrogen and stored at −80 °C for subsequent RNA and protein extraction; and (iii) the remaining tissues were fixed in 4% paraformaldehyde, paraffin-embedded, and sectioned into consecutive 4-μm slices for immunohistochemistry (IHC) and immunofluorescence (IF) analyses.

#### Immunohistochemistry and immunofluorescence

Paraffin-embedded sections were baked at 60 °C for 2 h, followed by standard deparaffinization and rehydration. Antigen retrieval was performed by heat induction in sodium citrate buffer (pH 6.0; Servicebio, G1202) using a microwave oven. Endogenous peroxidase activity was blocked by incubation with 3% hydrogen peroxide in methanol for 25 min at room temperature in the dark. Sections were then blocked with 5% bovine serum albumin (BSA; Servicebio, G5001) for 30 min.

Slides were incubated overnight at 4 °C in a humidified chamber with the following primary antibodies: rabbit anti-human CR1 monoclonal antibody (Abcam, ab235882; 1:200), mouse anti-human CD68 monoclonal antibody (Dako, M0814; 1:100), rabbit anti-human CD206 monoclonal antibody (Abcam, ab64693; 1:200), and mouse anti-human CD8A monoclonal antibody (Dako, M7103; 1:100). Phosphate-buffered saline (PBS) was used instead of the primary antibody as a negative control.

After washing with PBS, sections were incubated with the corresponding horseradish peroxidase (HRP)-conjugated secondary antibodies (goat anti-rabbit/mouse IgG; Servicebio, GB23303/GB23301) for 50 min at room temperature. Signals were developed using a 3,3’-diaminobenzidine (DAB) kit (Servicebio, G1211), followed by hematoxylin counterstaining (Servicebio, G1004), differentiation with acid alcohol, and bluing with ammonia water. Sections were dehydrated through graded ethanol, cleared in xylene, and mounted with neutral balsam (Servicebio, G1401).

For double immunofluorescence staining, a similar procedure was followed, except that fluorophore-conjugated secondary antibodies were used and nuclei were counterstained with 4’,6-diamidino-2-phenylindole (DAPI).

All stained slides were independently evaluated by two experienced pathologists blinded to **the** clinical and pathological information. Expression levels of CR1, CD68, and CD206 were semi-quantitatively assessed using the H-score system:

$${\text{H - score}} = \mathop \sum \nolimits {\left( {{P_i} \times i} \right)}$$

where Pi represents the percentage of cells stained at each intensity level (i = 0, negative; 1, weak; 2, moderate; 3, strong). Immunofluorescence results were quantified as the proportion of positive signal (positive area or positive cell percentage) [51].

#### Cell culture and induction of macrophage differentiation

The human monocytic cell line THP-1 (ATCC TIB-202) was cultured in RPMI-1640 medium (Gibco, 11,875,093) supplemented with 10% fetal bovine serum (FBS; Gibco, 10,270,106) and 1% penicillin-streptomycin (Gibco, 15,140,122) at 37 °C in a humidified incubator with 5% CO_2_.

To induce macrophage differentiation, cells were seeded at a density of 5 × 10^5 cells/mL and treated with 100 ng/mL phorbol 12-myristate 13-acetate (PMA; Sigma-Aldrich, P8139) for 48 h. The medium was then replaced with fresh complete medium, and cells were cultured for an additional 24 h. Adherent cells exhibiting macrophage-like morphology were defined as M0 macrophages and used for subsequent experiments [52].

#### Gene knockdown and overexpression

CR1 expression was manipulated using RNA interference and plasmid-mediated overexpression approaches. THP-1-derived macrophages were transfected with small interfering RNAs (siRNAs) targeting human CR1 or negative-control siRNA (si-NC) at a final concentration of 50 nM using Lipofectamine RNAiMAX (Invitrogen, 13,778,150), according to the manufacturer’s instructions.

For overexpression experiments, the full-length human CR1 coding sequence was cloned into the pcDNA3.1 expression vector, and cells were transfected using Lipofectamine 3000 (Invitrogen, L3000015), with the empty vector serving as the control. All siRNAs were synthesized by RiboBio (Guangzhou, China).

Cells were harvested 48 h after transfection, and knockdown or overexpression efficiency was confirmed by quantitative real-time PCR (qRT-PCR) and Western blotting. The sequences of all siRNAs used in this study are listed in Table 1.

**Table 1:** Sequences of siRnas targeting CR1

siRNA	Forward (5′–3′)	Reverse (5′–3′)
si-CR1#1	GCAACAUCAUUGAGCUCAATT	UUGAGCUCAUGAUGUUGCTT
si-CR1#2	CCUGAAGAUGGAGCAGUUUTT	AAACUGCUCCAUCUUCAGGTT
si-NC	UUCUCCGAACGUGUCACGUTT	ACGUGACACGUUCGGAGAATT

#### RNA extraction and qRT-PCR

Total RNA was extracted from cultured cells using TRIzol reagent (Invitrogen, 15,596,026) according to the manufacturer’s instructions. RNA concentration and purity were determined using a NanoDrop 2000 spectrophotometer (Thermo Scientific) by measuring the optical density at 260/280 nm. For complementary DNA (cDNA) synthesis, 1 μg of total RNA was treated with genomic DNA eraser and reverse-transcribed using the PrimeScript RT reagent Kit with gDNA Eraser (Takara, RR047A).

qRT-PCR was performed using TB Green Premix Ex Taq II (Tli RNaseH Plus) (Takara, RR820A) on a QuantStudio 6 Flex Real-Time PCR System (Applied Biosystems). Each 20-μL reaction mixture contained 10 μL of 2× TB Green Premix Ex Taq II, 0.8 μL of forward primer (10 μM), 0.8 μL of reverse primer (10 μM), 2 μL of cDNA template, and 6.4 μL of RNase-free water.

The amplification conditions were as follows: initial denaturation at 95 °C for 30 s; 40 cycles of 95 °C for 5 s and 60 °C for 30 s; followed by a melt-curve analysis from 60 °C to 95 °C to verify product specificity. Glyceraldehyde-3-phosphate dehydrogenase (GAPDH) was used as the internal reference gene. Relative gene expression levels were calculated using the 2^-ΔΔCt method [53]. The primer sequences used in this study are listed in Table 2.

**Table 2:** Sequences of primers used for qRT-PCR

Gene	Forward (5′–3′)	Reverse (5′–3′)
CR1	CACCATGGCCTCTGTGTCTA	GGCAGGTAGGTGTTGTCAGG
GAPDH	GGAGCGAGATCCCTCCAAAAT	GGCTGTTGTCATACTTCTCATGG

#### Western blot analysis

Cells were lysed on ice for 30 min using radioimmunoprecipitation assay (RIPA) buffer (Beyotime, P0013B) supplemented with 1 mM phenylmethylsulfonyl fluoride (PMSF; Beyotime, ST506) and a protease inhibitor cocktail (Roche, 4,693,132,001). Lysates were centrifuged at 12,000 rpm for 15 min at 4 °C, and the supernatants were collected. Protein concentrations were determined using a bicinchoninic acid (BCA) protein assay kit (Beyotime, P0010).

Equal amounts of protein (30 μg) were mixed with 6× loading buffer and denatured by boiling at 100 °C for 10 min. Proteins were separated by 8% sodium dodecyl sulfate-polyacrylamide gel electrophoresis (SDS-PAGE) and subsequently transferred onto polyvinylidene difluoride (PVDF) membranes (Millipore, IPVH00010) using a wet-transfer system at a constant current of 250 mA for 90 min. Membranes were blocked with Tris-buffered saline containing 0.1% Tween-20 (TBST) supplemented with 5% non-fat milk for 1 h at room temperature.

The membranes were incubated overnight at 4 °C with the following primary antibodies: rabbit anti-CR1 antibody (Abcam, ab235882; 1:1000) and mouse anti-β-actin antibody (Proteintech, 60,004–1-Ig; 1:5000). After three washes with TBST (10 min each), membranes were incubated with the corresponding HRP-conjugated goat anti-rabbit or anti-mouse secondary antibodies (Abcam, ab6721/ab6789; 1:5000) for 1 h at room temperature. Following extensive washing with TBST, protein bands were visualized using an enhanced chemiluminescence (ECL) detection kit (NCM Biotech, P10300) and imaged using a ChemiDoc MP Imaging System (Bio-Rad). Band intensities were quantified using ImageJ software (v1.53).

#### Macrophage polarization, phagocytosis and co-culture assays

To evaluate the effects of CR1 on macrophage polarization, genetically manipulated THP-1-derived macrophages were stimulated with either 100 ng/mL lipopolysaccharide (LPS; Sigma, L4391) plus 20 ng/mL interferon-gamma (IFN-gamma; PeproTech, 300–02) to induce M1 polarization, or 20 ng/mL interleukin-4 (IL-4; PeproTech, 200–04) plus 20 ng/mL interleukin-13 (IL-13; PeproTech, 200–13) to induce M2 polarization. After 24 h of stimulation, macrophage polarization status was assessed by flow-cytometric analysis of surface markers (CD86 for M1 and CD206 for M2) or by qRT-PCR analysis of polarization-related genes (tumor necrosis factor-alpha [TNF-alpha] for M1 and IL-10 for M2).

Macrophage phagocytic capacity was evaluated using fluorescein isothiocyanate (FITC)-labeled phagocytic particles incubated with macrophages for 2 h. The proportion of FITC-positive cells was quantified by flow cytometry as an indicator of phagocytic activity [54].

To investigate the effects of CR1-modulated macrophages on T-cell function, an in vitro co-culture system was established. CD8^+^ T cells were isolated from peripheral blood mononuclear cells (PBMCs) obtained from healthy donors using CD8 MicroBeads (Miltenyi Biotec, 130–045-201) and activated with anti-CD3/CD28 antibodies (1 μg/mL; Miltenyi Biotec, 130–093-387). Macrophages were directly co-cultured with activated CD8^+^ T cells at a ratio of 1:2 (macrophages:CD8^+^ T cells). After 72 h of co-culture, T cells were harvested and stained with carboxyfluorescein succinimidyl ester (CFSE; Invitrogen, C34554) to assess cell proliferation. Intracellular expression of effector molecules, including IFN-gamma and granzyme B, was subsequently analyzed by flow cytometry [55].

### Statistical analysis

For MR analyses, statistical significance was defined as *p* < 0.05 using the IVW method, and multiple testing was corrected using the false discovery rate (FDR) approach with the Benjamini-Hochberg procedure. All in vitro experimental data were obtained from at least three independent biological replicates and are presented as mean ± standard error of the mean (SEM).

For normally distributed data, comparisons between two groups were performed using a two-tailed Student’s t-test, whereas comparisons among multiple groups were conducted using one-way analysis of variance (ANOVA), followed by Tukey’s post hoc test when appropriate. Non-normally distributed data were analyzed using the Mann-Whitney U test (two groups) or the Kruskal-Wallis test (multiple groups). Correlation analyses were performed using Pearson’s correlation or Spearman’s rank correlation, depending on data distribution and variance homogeneity. All statistical analyses were conducted using R software (v4.2.0) or GraphPad Prism 9. A two-sided *p* < 0.05 was considered statistically significant.

## Results

### MR analysis of pQTLs and HCC

**Fig. 1:**
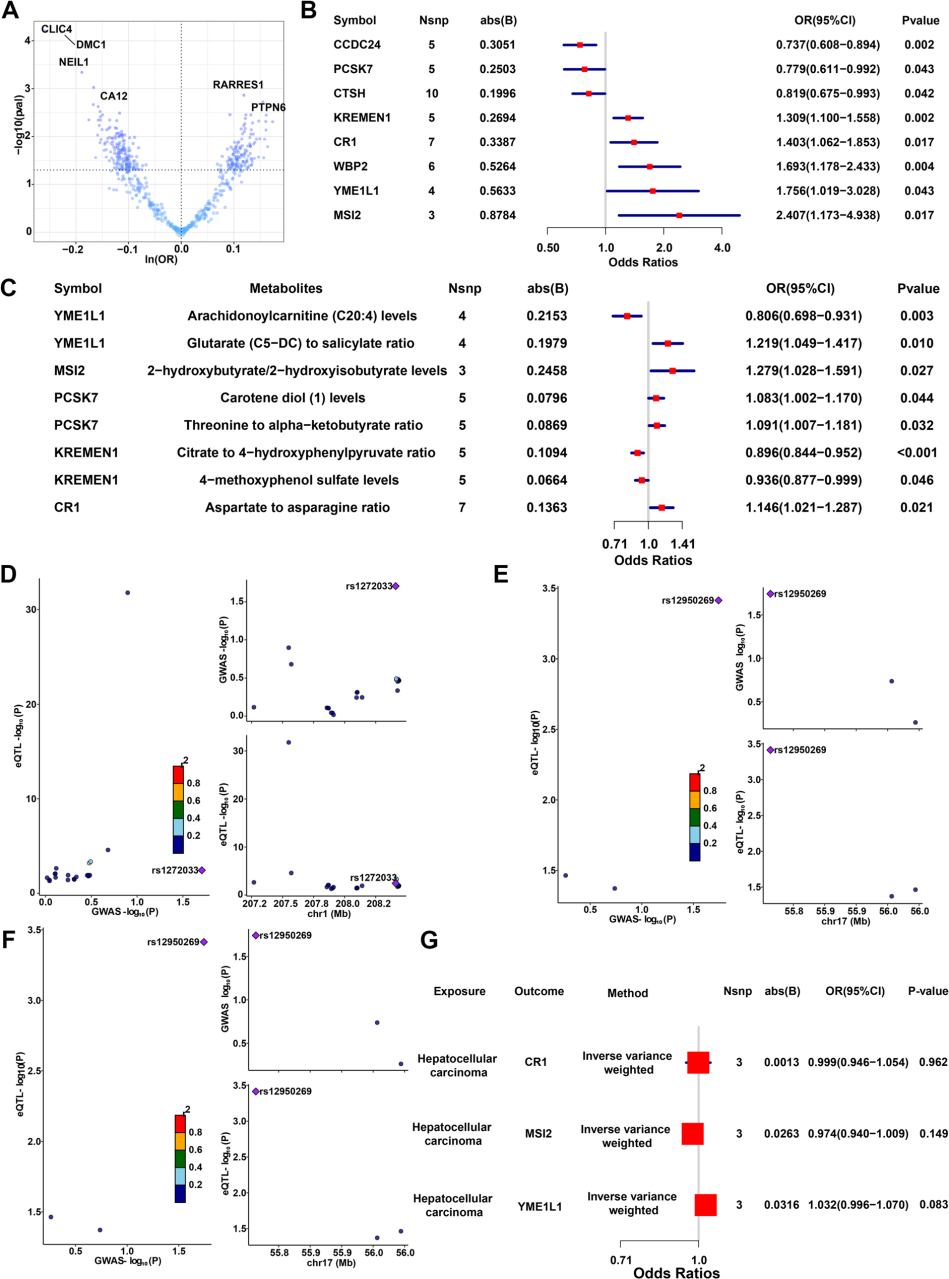
Mendelian randomization and colocalization analyses. (**A**) Distribution of ORs and *P* values for 316 causal associations identified by pQTL-based MR analysis. (**B**) Distribution of ORs and *P* values for eight causal associations identified by eQTL-based MR analysis. (**C**) Distribution of ORs and *p* values for eight causal associations between eQtls and metabolites identified by MR analysis. (**D**–**F**) Coloc analyses of CR1 (**D**), YME1L1 (**E**), and MSI2 (**F**), showing the regional association patterns between eQTL signals and HCC GWAS signals to evaluate whether they share common causal variants (SNP.PP.H4). (**G**) Reverse MR analysis using HCC as the exposure and CR1, MSI2, and YME1L1 expression as the outcomes, showing the distributions of ORs and *p* values for the reverse causal effects. MR, Mendelian randomization; pQTL, protein quantitative trait loci; eQTL, expression quantitative trait loci; HCC, hepatocellular carcinoma; GWAS, genome-wide association study; OR, odds ratio

Based on the HCC GWAS summary statistics (outcome ID: C3_HEPATOCELLU_CARC_EXALLC), a total of 545 pQTL-outcome associations were extracted using the extract_instruments and extract_outcome_data functions. MR analyses were subsequently performed using the IVW method, identifying 316 pQTL-associated genes with significant causal associations with HCC risk (Fig. 1A, IVW *p* < 0.05). The complete forest plots are provided in Supplementary Figure S1. Among these, 138 genes (e.g., BST2, DCN, and PGLYRP1) were associated with an increased risk of HCC, whereas 178 genes (e.g., CLIC4, DMC1, and NEIL1) were associated with a decreased risk of HCC. Sensitivity analyses showed that the overall causal estimates remained stable after sequential removal of individual SNPs, indicating that the results were not driven by any single instrument.

### MR analysis of eQTLs and HCC

To further identify key candidate genes, eQTL analyses were performed for the 316 pQTL-associated genes, resulting in the extraction of 10 eQTLs-outcome associations. Subsequent MR analyses identified eight genes that were significantly associated with HCC risk (Fig. 1B, IVW *p* < 0.05). Specifically, higher genetically predicted expression levels of KREMEN1, CR1, WBP2, YME1L1, and MSI2 were significantly associated with an increased risk of HCC (odds ratio [OR] = 1.309–2.407). In contrast, increased expression of CCDC24, PCSK7, and CTSH was associated with a reduced risk of HCC (OR = 0.737–0.819). Leave-one-out sensitivity analyses showed that the causal estimates remained consistent after the exclusion of each individual SNP in turn, confirming the robustness of these associations (Supplementary Fig. S2A-H).

### Mediation analysis: establishing a gene–metabolite–HCC causal axis

#### MR analysis of metabolites and HCC

To identify potential causal metabolites associated with HCC at the metabolic level, HCC GWAS summary statistics (outcome ID: C3_HEPATOCELLU_CARC_EXALLC) were used to extract metabolite-outcome pairs through the extract_instruments and extract_outcome_data functions. A total of 118 metabolite-outcome pairs were obtained. Two-sample Mendelian randomization (MR) analyses were subsequently performed to systematically evaluate the causal relationships between circulating metabolites and HCC risk. This analysis identified 64 metabolite-HCC pairs showing significant causal associations (IVW *p* < 0.05) (Supplementary Fig. S3). Among these, several metabolites were negatively associated with HCC risk, including the 3-methyl-2-oxovalerate to 4-methyl-2-oxopentanoate ratio, the serine-to-alpha-ketobutyrate ratio, 1-linoleoylglycerol (18:2), valine, and phosphoethanolamine. In contrast, other metabolites, such as arachidonoylcarnitine (C20:4), 2-hydroxybutyrate/2-hydroxyisobutyrate, cystathionine, and the aspartate-to-asparagine ratio, were positively associated with increased HCC risk.

Leave-one-out sensitivity analyses showed that sequential removal of any single SNP did not materially alter the overall effect estimates or confidence intervals, indicating that the 64 identified metabolite-HCC causal associations were robust and reliable.

#### MR analysis of eQTLs and metabolites

To further elucidate how genetic regulation may influence HCC development through metabolic pathways, the eight significant eQTL-associated genes identified above were paired as exposures with the 64 HCC-related metabolites as outcomes. A total of 25 eQTL-metabolite pairs were extracted. Two-sample MR analyses identified eight significant causal associations (IVW *p* < 0.05) (Fig. 1C). These causal links primarily involved five key genes (YME1L1, MSI2, PCSK7, KREMEN1, and CR1) and eight metabolites, including arachidonoylcarnitine (C20:4), 2-hydroxybutyrate/2-hydroxyisobutyrate, carotene diol (1), the threonine to alpha-ketobutyrate ratio, the citrate to 4-hydroxyphenylpyruvate ratio, 4-methoxyphenol sulfate, the aspartate to asparagine ratio, and the glutarate (C5-DC) to salicylate ratio.

Leave-one-out sensitivity analyses further demonstrated that the overall effect directions and confidence intervals remained stable after sequential exclusion of individual SNPs, with no substantial deviations observed, supporting the robustness of the identified eQTL-metabolite causal relationships (Supplementary Fig. S4A-H).

#### Metabolite-mediated effects and reverse causality analyses

After establishing the causal relationships between eQTLs and metabolites, as well as between metabolites and HCC, we next investigated the mediating roles of metabolites in the gene-HCC axis. The results indicated that 2-hydroxybutyrate/2-hydroxyisobutyrate, the C5-DC to salicylate ratio, and the aspartate to asparagine ratio may serve as key mediators linking CR1, YME1L1, and MSI2 to HCC risk. Colocalization analyses performed using coloc demonstrated strong evidence of shared causal variants at the eQTL-disease level for these three genes (SNP.PP.H4 > 0.95) (Fig. 1D–F), indicating that the same genetic variants jointly influence gene expression and disease susceptibility, thereby supporting a common genetic architecture. To further validate the directionality and robustness of the inferred causal relationships, reverse Mendelian randomization analyses were conducted for the three genes (Fig. 1G). No significant reverse causal effects were observed for CR1, MSI2, or YME1L1 (*p* = 0.962, 0.149, and 0.083, respectively), providing additional support for a unidirectional causal pathway from genetically regulated gene expression to metabolic alterations and ultimately to HCC development.

### Clinical relevance and drug response analyses of key genes

#### Associations between key gene expression and chemotherapeutic drug sensitivity

Based on the GDSC database, drug response in tumor samples was predicted using the pRRophetic R package, and correlations between the expression levels of the key genes (CR1, YME1L1, and MSI2) and IC50 values were analyzed. The results showed that expression of the key genes was significantly associated with the predicted IC50 values of multiple anticancer agents (Supplementary Fig. S5A-F). Specifically, high expression of CR1, YME1L1, and MSI2 was significantly associated with increased sensitivity (lower IC50 values) to bleomycin, bosutinib, and doxorubicin (Supplementary Fig. S5A, C, F). In contrast, for bortezomib, bryostatin-1, and dasatinib, the relationships between gene expression and IC50 values showed drug- and gene-specific patterns (Supplementary Fig. S5B, D, E). Collectively, these findings suggest that the expression status of these key genes may serve as a potential predictor of chemotherapeutic response in HCC.

#### Associations between key gene expression and clinicopathological features

To evaluate the clinical relevance of the key genes, clinicopathological characteristics from the TCGA-HCC cohort, including age, sex, tumor stage, T stage, N stage, and M stage, were systematically analyzed. Using boxplot visualization and appropriate statistical tests (Student’s t-test or Kruskal-Wallis test), we found that the expression levels of CR1, YME1L1, and MSI2 were all significantly associated with tumor stage and T stage (*p* < 0.05) (Supplementary Fig. S6A-X). Notably, CR1 expression showed strong positive correlations with tumor stage (*p* = 4.5 × 10^-3), tumor grade (*p* < 1.0 × 10^-4), T stage (*p* = 9.8 × 10^-3), and N stage (*p* = 2.72 × 10^-2). In advanced-stage (III-IV) or highly invasive (T3–T4) tumors, the expression levels of these three genes were consistently elevated, indicating that they may play important roles in HCC progression and invasiveness.

#### Molecular docking analyses validating the potential interactions of CR1, YME1L1, and MSI2 with dasatinib

Given that drug-sensitivity analyses indicated that dasatinib, a multi-target tyrosine kinase inhibitor, exhibited significant IC50 differences between the high- and low-expression groups of CR1, YME1L1, and MSI2, we further explored the potential binding modes between dasatinib and these proteins at the structural level. Protein-compound pairs were defined as follows: CR1 (P17927)-dasatinib, YME1L1 (Q96TA2)-dasatinib, and MSI2 (Q96DH6)-dasatinib. Docking binding energies are summarized in Supplementary Table S1, and representative docking conformations are shown in Supplementary Fig. S7A-C. The predicted binding energies were −7.0 kcal/mol for CR1-dasatinib, −7.3 kcal/mol for YME1L1-dasatinib, and −6.3 kcal/mol for MSI2-dasatinib. Dasatinib was predicted to stably occupy the binding pockets of CR1, YME1L1, and MSI2, forming multiple hydrogen bonds and hydrophobic interactions. These results suggest potential direct interactions between dasatinib and these proteins and provide structural support for their candidacy as therapeutic targets.

### Key gene expression reshapes the association patterns between the intratumoral microbiome and the immune microenvironment

**Fig. 2:**
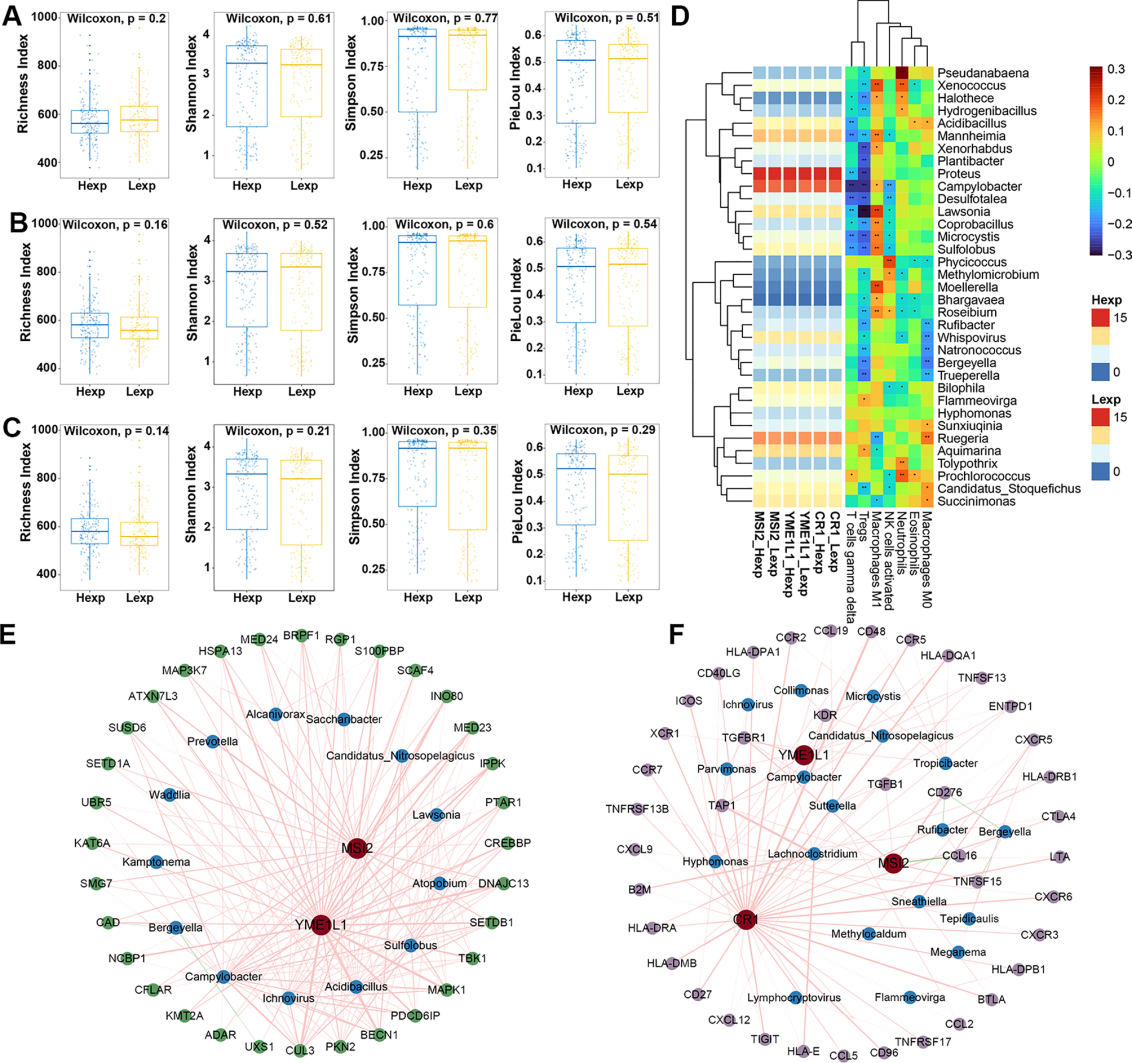
Associations between the intratumoral microbiome and the immune microenvironment. (**A**–**C**) Comparison of microbial α-diversity indices (richness, Shannon, Simpson, and Pielou) between high- and low-expression groups of key genes (CR1, MSI2, and YME1L1). *p* values were calculated using the Wilcoxon test and are indicated in the plots. (**D**) Heatmap showing Pearson correlation analysis between microbial genus abundance and immune cell infiltration levels. Red indicates positive correlations and blue indicates negative correlations. Neutrophils, eosinophils, and M0 macrophages were positively correlated with genera including prochlorococcus, succinimonas, and acidibacillus, whereas γδ T cells, Tregs, and activated NK cells were negatively correlated with genera including Campylobacter, Desulfotalea, Lawsonia, coprobacillus, microcystis, and sulfolobus. (**E**) Network showing the associations between CTL evasion–related genes (mainly MSI2 and YME1L1) and specific microbial taxa. (**F**) Network illustrating the broader and more complex interaction landscape between immunomodulatory genes (CR1, YME1L1, and MSI2) and tumor-resident microbes. CTL, cytotoxic T lymphocyte; Tregs, regulatory T cells

To investigate the roles of the key genes (CR1, YME1L1, and MSI2) within the tumor microenvironment, we analyzed the relationships between their expression levels and intratumoral microbial features. The results showed that expression of the key genes did not significantly alter overall microbial alpha-diversity (Fig. 2A–C) but markedly influenced interactions between the microbiome and the immune system. Specifically, the abundances of certain intratumoral bacterial genera were broadly correlated with immune-cell infiltration levels (Fig. 2D). For example, Prochlorococcus and Succinimonas were positively correlated with subsets of innate immune cells, whereas Campylobacter and Desulfotalea were negatively correlated with adaptive immune populations, including gamma-delta T cells and Tregs.

Further network analyses demonstrated that CTL immune-escape-related genes were primarily embedded within interaction networks involving specific microbial clusters (Fig. 2E), whereas immunoregulatory genes exhibited broader and more complex microbial interaction patterns (Fig. 2F). These findings indicate that functionally distinct key genes may participate in regulation of the tumor microenvironment through differential microbiome-immune coupling pathways.

### Single-cell and spatial transcriptomic analyses reveal the cellular specificity and spatial heterogeneity of key genes

To systematically characterize the expression patterns of the key genes (CR1, YME1L1, and MSI2) in the HCC microenvironment at both the cellular and tissue levels, we integrated scRNA-seq and spatial transcriptomic datasets.

#### Single-cell level: key genes exhibit cell type–specific expression patterns

After rigorous quality control of high-quality single-cell data comprising 53,474 cells, the distributions and correlations of nCount_RNA, nFeature_RNA, percent.mt, and percent.ribo across samples were visualized (Supplementary Fig. S8A, B). A total of 2,000 highly variable genes were identified (Supplementary Fig. S8D), and the data were subsequently normalized and subjected to dimensionality reduction. PCA and Elbow plots were used to determine the major principal components (Supplementary Fig. S8C, E), followed by batch-effect correction using the Harmony algorithm (Supplementary Fig. S8F).

**Fig. 3:**
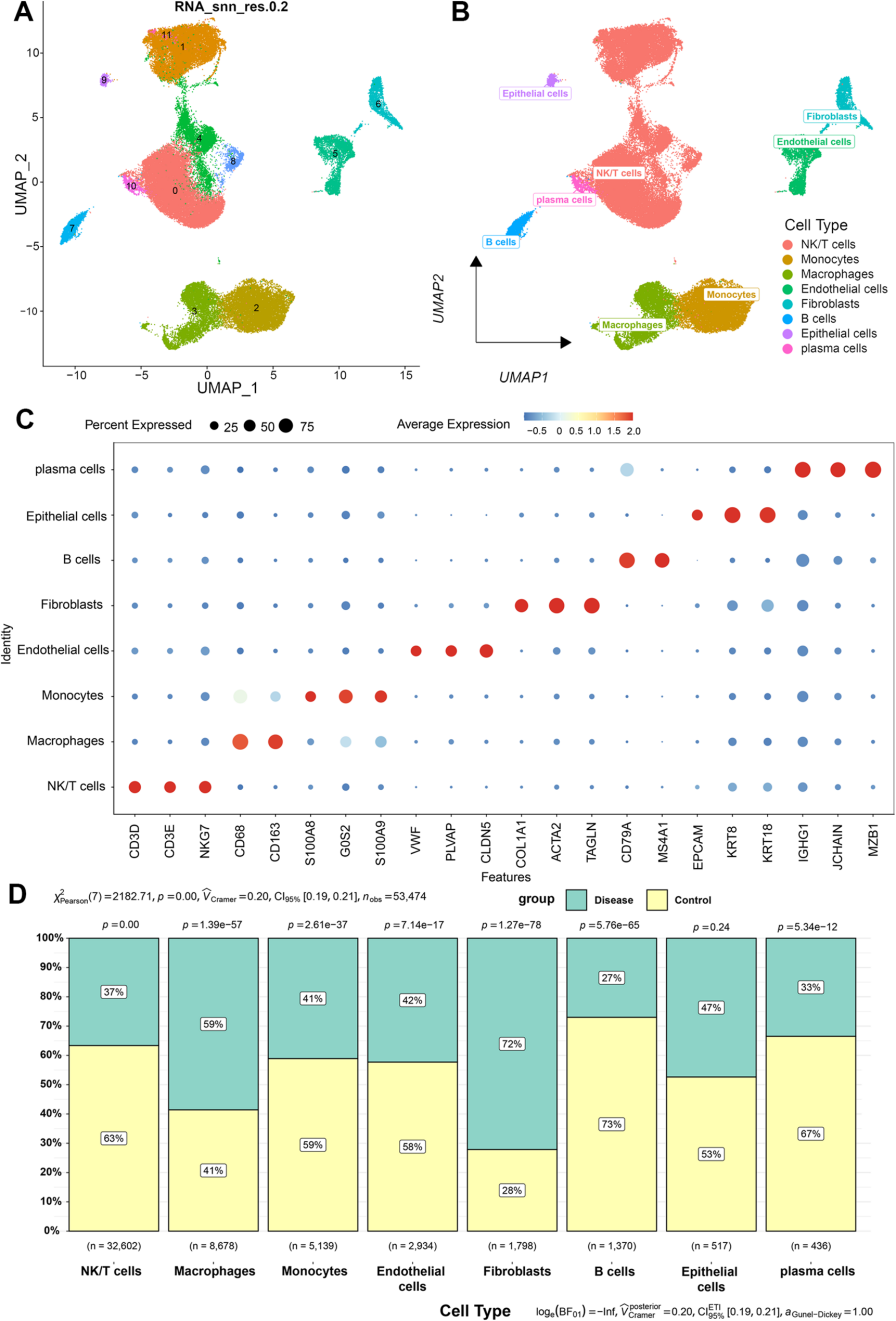
Single-cell annotation of major cell populations in HCC. (**A**) UMAP visualization of single cells based on PCA, identifying 12 distinct clusters. (**B**) Cell-type annotation of the 12 clusters, which were classified into eight major cell populations: NK/T cells, macrophages, monocytes, endothelial cells, fibroblasts, B cells, epithelial cells, and plasma cells. (**C**) Dot plot showing the expression patterns of canonical marker genes across the eight annotated cell types. (**D**) Proportional distribution of the eight major cell populations across the two sample groups. HCC, hepatocellular carcinoma; PCA, principal component analysis; UMAP, Uniform Manifold Approximation and Projection; NK, natural killer

UMAP analysis identified 12 distinct cellular clusters (Fig. 3A), which were annotated into eight major cell types, including natural killer (NK)/T cells, macrophages, monocytes, endothelial cells, fibroblasts, B cells, epithelial cells, and plasma cells (Fig. 3B). The expression patterns of canonical marker genes for each cell type are shown in Fig. 3C, and their proportional distributions are summarized in Fig. 3D, with fibroblasts exhibiting the greatest inter-sample variability.

**Fig. 4:**
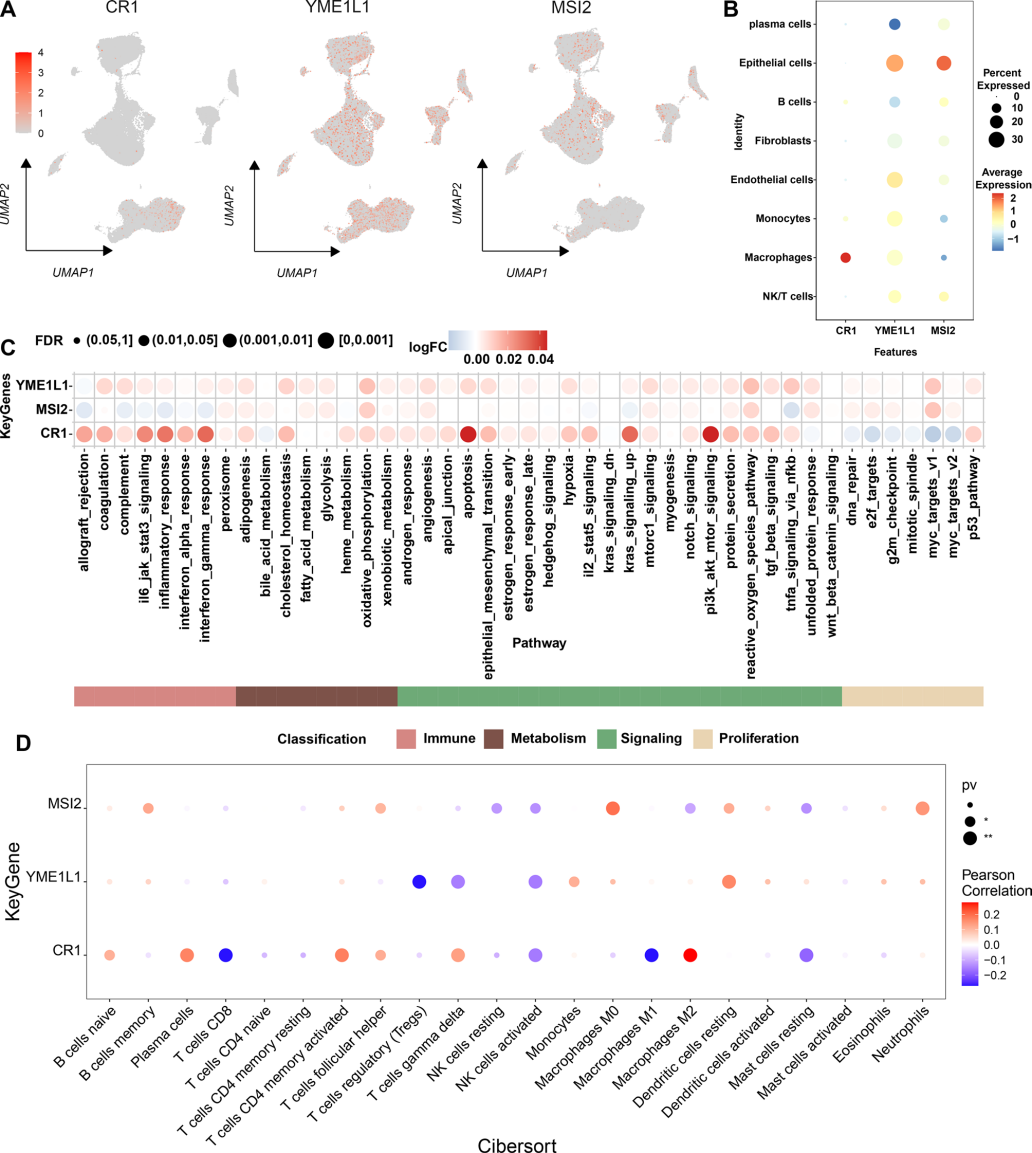
Single-cell expression patterns of key genes and associated immunometabolic pathway activities. (**A**) Scatter plot showing the overall expression patterns of key genes at the single-cell level. (**B**) Bubble plot depicting the expression of key genes across different cell types; bubble size represents the proportion of expressing cells, and color indicates the average expression level (red, high expression; blue, low expression). (**C**) Heatmap showing differences in immune- and metabolism-related pathway activities associated with key genes, with red indicating higher pathway activity and blue indicating lower activity. (**D**) Correlation analysis between key gene expression levels and immune cell infiltration; bubble color represents the direction and strength of correlation (Pearson’s correlation coefficient; red, positive; blue, negative), and bubble size reflects statistical significance (*P* value). Immune cell infiltration was estimated using CIBERSORT. Key genes include CR1, YME1L1, and MSI2. PCA, principal component analysis; UMAP, Uniform Manifold Approximation and Projection

The key genes displayed markedly distinct expression preferences across cell populations (Fig. 4A, B). CR1 showed highly cell-type-specific expression and was predominantly enriched in macrophages and monocytes. MSI2 showed a broader expression pattern, with relatively high expression in epithelial cells and subsets of stromal and myeloid cells, whereas YME1L1 showed the most ubiquitous expression and was detected across nearly all identified cell types.

Pathway activity analyses revealed that, compared with MSI2 and YME1L1, high CR1 expression was significantly enriched in multiple immune- and metabolism-related pathways, including complement activation, interferon-gamma response, inflammatory response, JAK-STAT3 signaling, TNF-alpha signaling, KRAS signaling, and apoptosis, underscoring a central role for CR1 in remodeling the tumor immune microenvironment (Fig. 4C).

Furthermore, immune-infiltration analysis using CIBERSORT demonstrated that CR1 expression was strongly positively correlated with M2 macrophage infiltration while being significantly negatively correlated with CD8^+^ T cells, NK cells, and M1 macrophages (Fig. 4D). In contrast, MSI2 and YME1L1 were more closely associated with NK cells, monocytes, and subsets of adaptive immune-cell populations. Collectively, these findings highlight CR1 as a pivotal regulatory factor that may promote formation of an immunosuppressive microenvironment by coordinating innate and adaptive immune responses.

#### Spatial transcriptomic level: key genes display distinct spatial heterogeneity

**Fig. 5:**
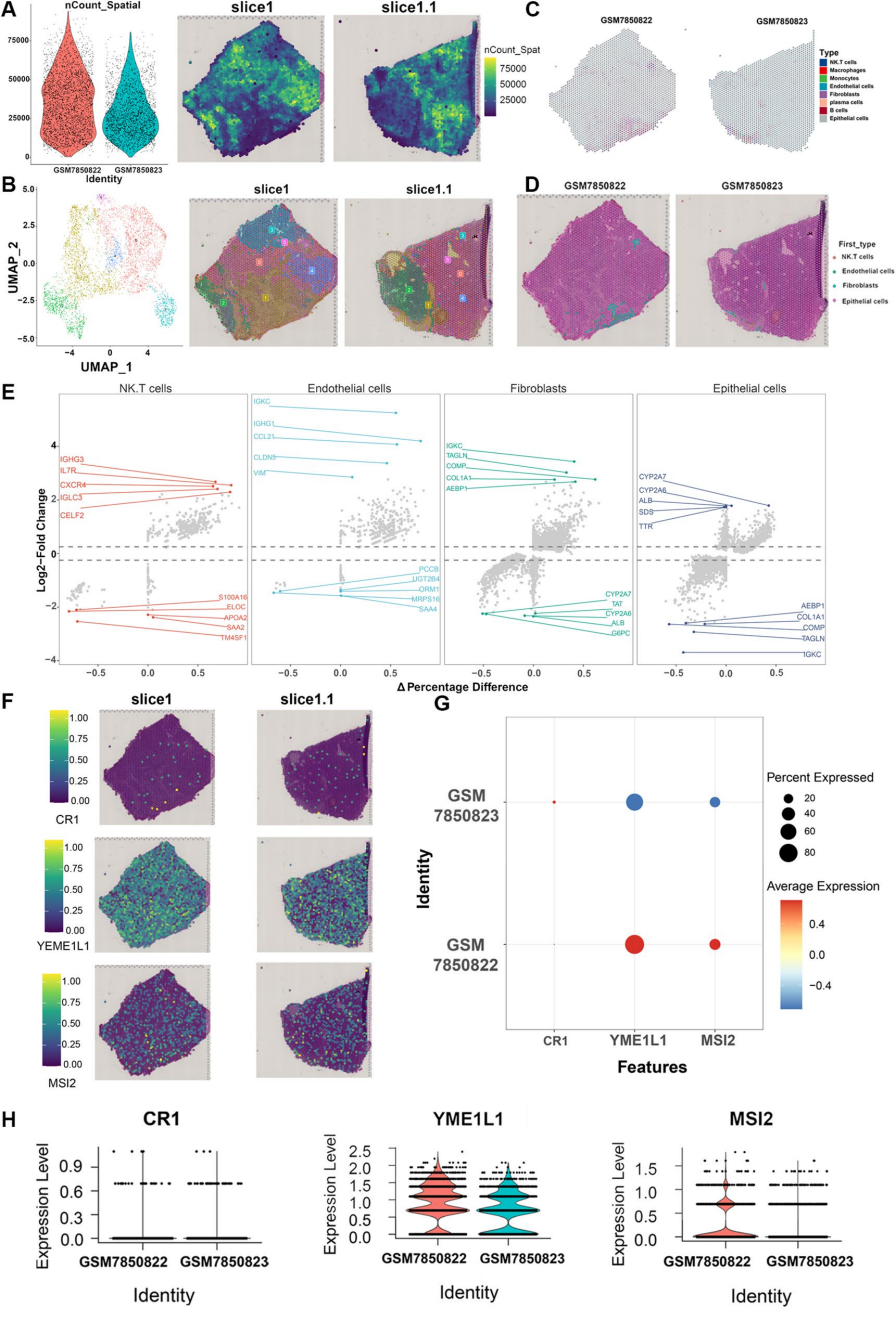
Spatial transcriptomic profiling reveals cellular architecture in hepatocellular carcinoma. (**A**) Spatial distribution of nCount_spatial (unique molecular identifier [UMI] counts) across two spatial transcriptomic sections; regions with higher UMI counts generally correspond to epithelial-enriched areas. (**B**) UMAP visualization showing six distinct cellular clusters identified by unsupervised Louvain clustering. (**C**) Deconvolution results based on the spacexr package, illustrating the proportional composition of different cell types within each spatial spot. (**D**) Spatial cell-type map assigning each spot according to its dominant cell type. (**E**) Distribution of differentially expressed marker genes across major cell types, including log2FC and differences in cell-type proportions, supporting the reliability of cell-type annotation. (**F**) Scatter plots showing the spatial expression patterns of key genes. (**G**) Bubble plots showing the spatial expression abundance of key genes; bubble size represents the proportion of expressing spots, and color indicates the average expression level (blue, high expression; red, low expression). (**H**) Spatial expression distributions of CR1, YME1L1, and MSI2 across two HCC sections. ST, spatial transcriptomics; UMAP, Uniform Manifold Approximation and Projection; Log2FC, log2 Fold chang

Spatial transcriptomic analyses revealed pronounced spatial heterogeneity and cellular architectural organization within HCC tissues. After data normalization and dimensionality reduction, six distinct spatial clusters were identified through unsupervised clustering (Fig. 5A, B). To resolve the cellular composition of each spatial spot, RCTD-based deconvolution was applied, enabling inference of cell-type proportions at each spatial location and construction of high-resolution spatial maps of cellular distribution (Fig. 5C, D). Expression patterns of canonical marker genes across spatial domains further validated the reliability of the deconvolution results (Fig. 5E). The key genes CR1, YME1L1, and MSI2 exhibited markedly distinct spatial expression patterns (Fig. 5F–H). YME1L1 showed the most widespread and highest-intensity expression, MSI2 displayed relatively broad but moderate expression, whereas CR1 showed a highly restricted and spatially localized enrichment pattern.

These findings provide direct evidence that CR1 is specifically localized to discrete spatial niches within the tumor microenvironment, suggesting that CR1 may exert unique regulatory functions by acting on specific cellular assemblies or microanatomical domains within the local tumor ecosystem.

### Clinical validation: CR1 is highly expressed in tumor tissues and associated with an immunosuppressive microenvironment

#### CR1 is specifically overexpressed in HCC tumor tissues

**Fig. 6:**
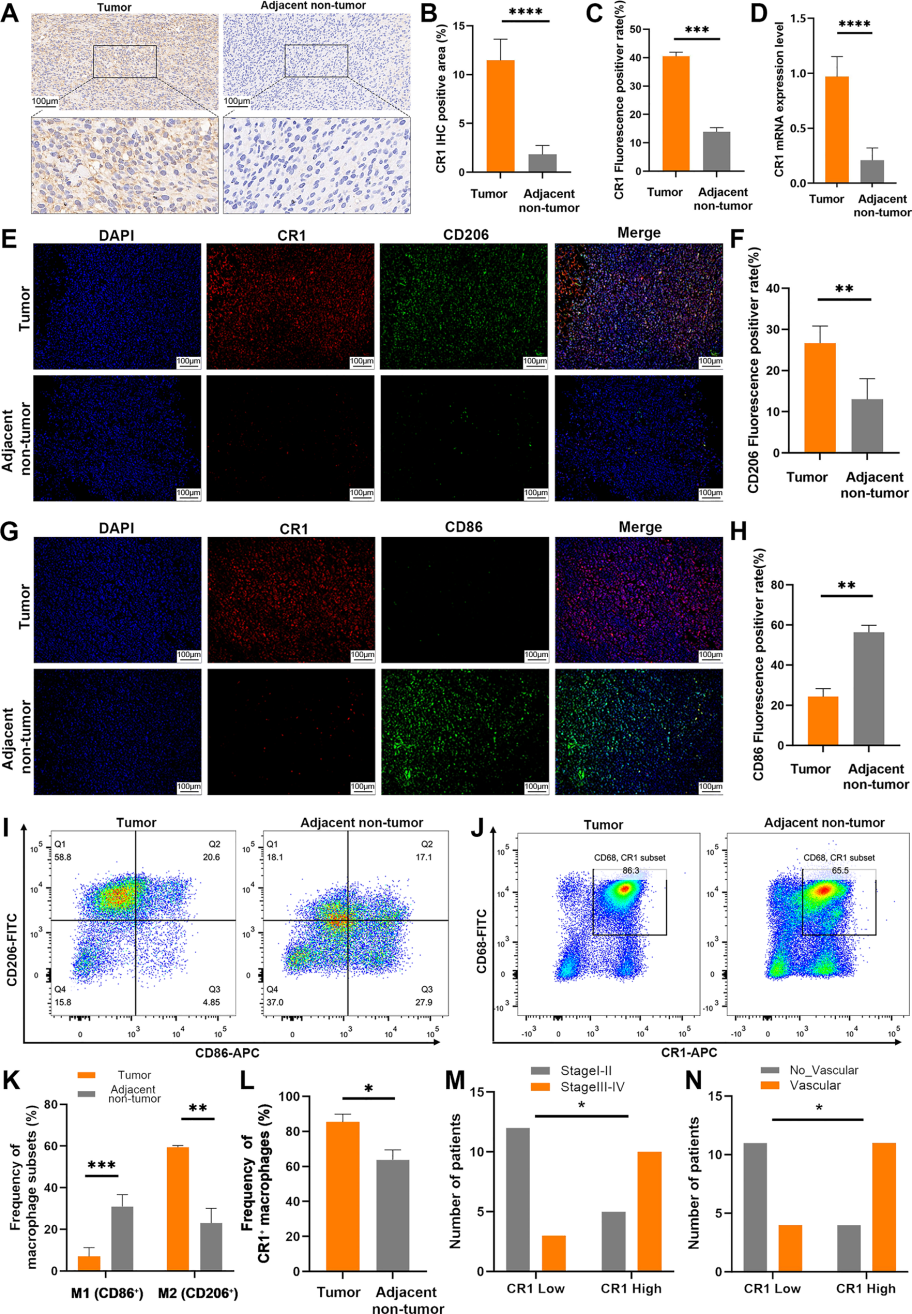
CR1 is upregulated in tumor tissues and associated with M2-like macrophage enrichment. (**A**) Representative IHC images of CR1 expression in HCC tissues and paired adjacent non-tumorous tissues. (**B**) Quantification of CR1-positive staining area in tumor and adjacent tissues (****p < 0.0001). (**C**) Quantitative analysis of CR1-positive immunofluorescence signals in tumor and adjacent tissues (***p < 0.001). (**D**) Relative mRNA expression levels of CR1 in tumor and adjacent tissues validated by qRT-PCR (****p < 0.0001). (**E**) Representative double immunofluorescence staining images showing colocalization of CR1 with CD206 (an M2 macrophage marker). (**F**) Quantitative analysis of CD206^+^ M2-like macrophage immunofluorescence signals (**p < 0.01). (**G**) Representative double immunofluorescence staining images showing CR1 and CD86 (an M1 macrophage marker). (**H**) Quantitative analysis of CD86^+^ M1-like macrophage immunofluorescence signals (**p < 0.01). (**I**) Representative flow cytometry plots showing CD206^+^ M2-like and CD86^+^ M1-like macrophages in tumor and adjacent tissues. (**J**) Representative flow cytometry plots showing CR1^+^ cells among CD68^+^ macrophages in tumor and adjacent tissues. (**K**) Statistical analysis of the proportions of M1 (CD86^+^) and M2 (CD206^+^) macrophages in tumor and adjacent tissues (**p < 0.01, ***p < 0.001). (**L**) Statistical analysis of the proportion of CR1^+^ cells among CD68^+^ macrophages in tumor and adjacent tissues (*p < 0.05). (**M**) Distribution of tumor stages (stage I-II vs. Stage III-IV) between patients with high (*n* = 15) and low (*n* = 15) CR1 expression (*p < 0.05). (N) Distribution of vascular invasion between patients with high (*n* = 15) and low (*n* = 15) CR1 expression (*p < 0.05). Quantitative data are presented as mean ± SEM where applicable, and error bars indicate SEM

To validate the expression profile of CR1, paired HCC tumor tissues and adjacent non-tumorous liver tissues were analyzed by IHC, IF, and qRT-PCR. IHC staining demonstrated that CR1 expression intensity was markedly higher in tumor tissues than in adjacent tissues (Fig. 6A). Quantitative analysis further confirmed a significant increase in both the CR1-positive staining area and staining intensity of CR1 in tumor samples (Fig. 6B). Consistently, IF analysis revealed a significantly elevated CR1 fluorescence signal in tumor tissues (Fig. 6C), and qRT-PCR showed that CR1 mRNA expression levels were significantly higher in tumor tissues than in paired adjacent tissues (Fig. 6D). Collectively, these results demonstrate that CR1 is specifically overexpressed within the HCC tumor microenvironment.

#### High CR1 expression is associated with M2 macrophage enrichment and reduced CD8^+^ T-cell infiltration

IF co-staining analyses revealed the spatial relationships between CR1 expression and macrophage subsets. In tumor tissues, CR1 signals showed significant co-localization with the M2 macrophage marker CD206, whereas an inverse and mutually exclusive spatial distribution was observed with the M1 macrophage marker CD86 (Fig. 6E, G). Quantitative analyses demonstrated that the proportion of CD206^+^ M2 macrophages was significantly higher in tumor tissues than in adjacent non-tumorous tissues, whereas the proportion of CD86^+^ M1 macrophages was reduced (Fig. 6F, H). Furthermore, based on 30 surgically resected HCC specimens, patients were stratified into CR1-high (*n* = 15) and CR1-low (*n* = 15) groups according to the median IHC H-score for CR1 in tumor tissues. Compared with the CR1-low group, patients with high CR1 expression exhibited significantly higher proportions of advanced-stage disease (stage III-IV) and vascular invasion (Fig. 6M, N). These findings indicate that high CR1 expression is not only closely associated with an immunosuppressive microenvironment but also significantly correlated with more aggressive tumor phenotypes.

**Fig. 7:**
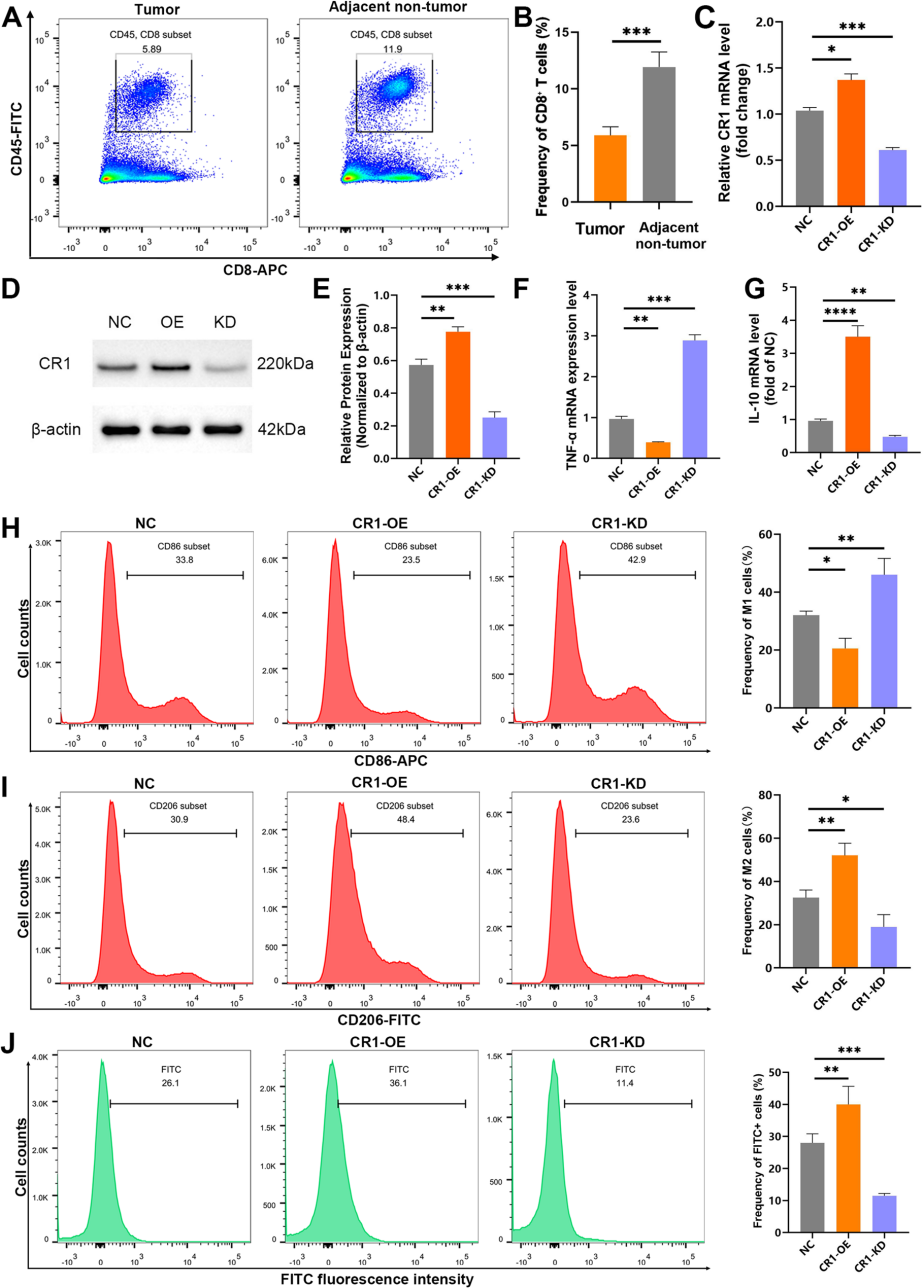
CR1 manipulation drives macrophage polarization and suppresses CD8^+^ T-cell function. (**A**) Representative flow cytometry plots of CD45^+^CD8^+^ T cells in hepatocellular carcinoma and adjacent non-tumorous tissues. (**B**) Statistical analysis of the proportion of CD8^+^ T cells among CD45^+^ leukocytes in tumor and adjacent tissues (***p < 0.001). (**C**) qRT-PCR analysis of CR1 mRNA expression in THP-1-derived M0 macrophages under different conditions (NC, CR1 overexpression [CR1-OE], and CR1 knockdown [CR1-KD]) (*p < 0.05, ***p < 0.001). (**D**) Representative Western blot images showing CR1 protein expression in different treatment groups. (**E**) Densitometric quantification of CR1 protein expression normalized to beta-actin (**p < 0.01, ***p < 0.001). (**F**) qRT-PCR analysis of TNF-alpha mRNA expression (**p < 0.01, ***p < 0.001). (**G**) qRT-PCR analysis of IL-10 mRNA expression (**p < 0.01, ****p < 0.0001). (**H**) Representative flow cytometry histograms and quantitative analysis of CD86^+^ M1-like macrophages (*p < 0.05, **p < 0.01). (**I**) Representative flow cytometry histograms and quantitative analysis of CD206^+^ M2-like macrophages (*p < 0.05, **p < 0.01). (**J**) Representative flow cytometry plots and quantitative analysis of macrophage phagocytic activity (**p < 0.01, ***p < 0.001). Quantitative data are presented as mean ± SEM from at least three independent biological replicates, and error bars indicate SEM. CR1, complement receptor 1; qRT-PCR, quantitative real-time polymerase chain reaction; OE, overexpression; KD, knockdown; NC, negative control

To further validate these observations at the single-cell level, flow-cytometric analyses were performed. The results showed that the proportion of CD206^+^ M2-like macrophages was significantly increased in tumor tissues compared with adjacent tissues (Fig. 6I, K), and the percentage of CR1^+^ cells within the macrophage population was also markedly elevated (Fig. 6J, L), consistent with the spatial immunofluorescence findings. In parallel, the proportion of CD8^+^ T cells among CD45^+^ leukocytes was significantly decreased in tumor tissues (Fig. 7A, B), indicating insufficient infiltration of cytotoxic T lymphocytes. Collectively, these clinical sample-based data demonstrate that high CR1 expression is tightly associated with M2 macrophage enrichment and reduced CD8^+^ T-cell infiltration, jointly defining an immunosuppressive tumor microenvironment.

### CR1 drives macrophage M2 polarization and suppresses CD8^+^ T-cell function

#### Validation of CR1 overexpression and knockdown efficiency

To verify the efficiency of CR1 genetic manipulation, THP-1-derived M0 macrophages were used to establish three experimental groups: negative control (NC), CR1 overexpression (OE), and CR1 knockdown (KD). qRT-PCR analysis showed that CR1 mRNA expression was significantly increased in the OE group and markedly reduced in the KD group compared with the NC group (Fig. 7C). Consistently, Western blot analysis demonstrated a pronounced upregulation of CR1 protein in the OE group and a significant downregulation in the KD group, in accordance with the mRNA expression changes (Fig. 7D). Densitometric quantification further confirmed that these differences were statistically significant (Fig. 7E). Collectively, these results confirm the successful establishment of CR1 overexpression and knockdown macrophage models at both the transcriptional and protein levels.

#### CR1 promotes macrophage M2 polarization and enhances phagocytic function

Following successful establishment of the CR1-manipulated macrophage models, we next evaluated the effects of CR1 on macrophage polarization phenotypes and functional properties. qRT-PCR analysis showed that, compared with the NC group, mRNA expression of the M2-associated marker IL-10 was significantly upregulated in the CR1 OE group and markedly reduced in the CR1 KD group. In contrast, the M1-associated marker TNF-α was downregulated in the OE group and upregulated in the KD group (Fig. 7F, G), indicating that CR1 expression drives macrophage polarization toward an M2 phenotype. Flow-cytometric analysis of surface markers further demonstrated that CR1 overexpression significantly increased the proportion of CD206^+^ M2 macrophages while reducing the proportion of CD86^+^ M1 macrophages, whereas CR1 knockdown produced the opposite effects (Fig. 7H, I). Functional assays revealed that CR1 overexpression markedly enhanced macrophage phagocytic capacity, whereas CR1 knockdown significantly impaired this function (Fig. 7J), indicating that CR1 not only regulates macrophage polarization status but also directly modulates macrophage effector functions.

#### CR1^+^ macrophages suppress CD8^+^ T-cell proliferation and cytotoxic function

**Fig. 8:**
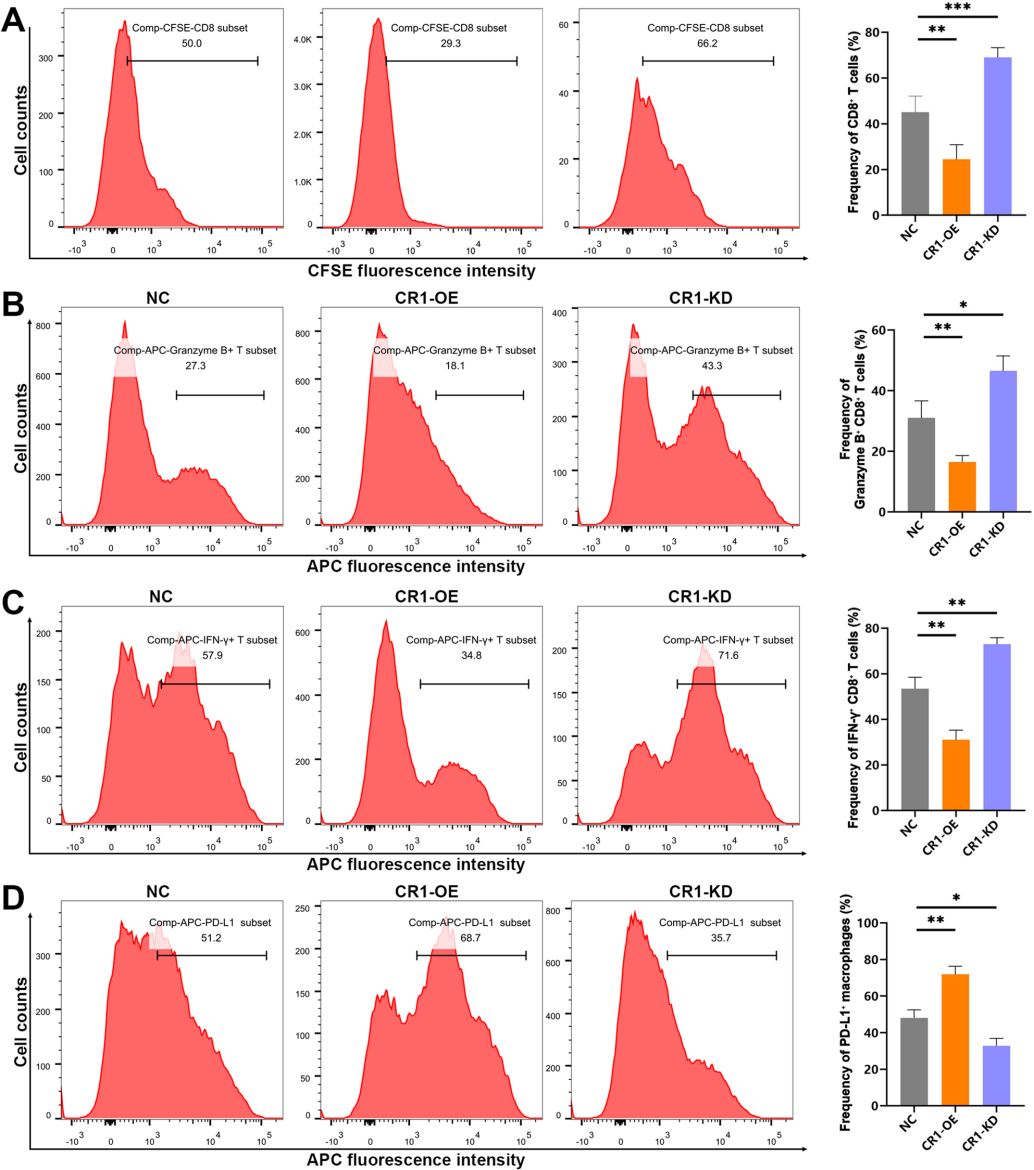
CR1^+^ macrophages suppress CD8^+^ T-cell proliferation and cytotoxic function. (**A**) Representative flow cytometry histograms and quantitative analysis of CD8^+^ T-cell proliferation assessed by CFSE dilution in the co-culture system (**p < 0.01, ***p < 0.001). (**B**) Representative flow cytometry plots and quantitative analysis of granzyme B^+^ CD8^+^ T cells (*p < 0.05, **p < 0.01). (**C**) Representative flow cytometry plots and quantitative analysis of IFN-gamma^+^ CD8^+^ T cells (**p < 0.01). (**D**) Representative flow cytometry plots and quantitative analysis of PD-L1 expression on macrophages in the co-culture system (*p < 0.05, **p < 0.01). Quantitative data are presented as mean ± SEM from at least three independent biological replicates. CFSE, carboxyfluorescein succinimidyl ester; IFN-γ, interferon-γ; PD-L1, programmed death-ligand 1

To clarify the regulatory effects of CR1^+^ macrophages on CD8^+^ T-cell function, an in vitro co-culture system was established, and T-cell proliferation and effector-molecule expression were assessed. CFSE dilution assays showed that, compared with the NC group, CR1-overexpressing macrophages significantly suppressed CD8^+^ T-cell proliferation, whereas CR1 knockdown markedly enhanced T-cell proliferation (Fig. 8A).

With respect to effector function, the proportion of IFN-γ^+^ CD8^+^ T cells was significantly reduced in the CR1 overexpression group and increased in the CR1 knockdown group. Similarly, the percentage of granzyme B^+^ CD8^+^ T cells was decreased in the OE group and elevated in the KD group (Fig. 8B, C). In the co-culture system, PD-L1 expression in macrophages was significantly upregulated in the CR1 overexpression group and downregulated in the CR1 knockdown group (Fig. 8D). Collectively, these results indicate that CR1 mediates macrophage-induced suppression of CD8^+^ T-cell function, at least in part through upregulation of PD-L1.

## Discussion

This study identifies CR1 as a previously underappreciated regulator of immune suppression in HCC and provides convergent evidence from genetics, transcriptomics, spatial mapping, clinical specimens, and functional assays. Several aspects distinguish the present work from prior studies. Rather than inferring TAM biology solely from expression correlations, we triangulated evidence from MR-based causal inference, metabolite mediation, single-cell and spatial localization, and perturbation experiments to show that CR1 is not merely a marker of macrophage infiltration but a plausible driver of an immunosuppressive macrophage state. Accordingly, the main contribution of this study is the establishment of a mechanistically coherent CR1-centered axis linking inherited susceptibility, macrophage polarization, and defective CD8^+^ T-cell immunity in HCC.

Our genetic analyses add a new dimension to current understanding of complement biology in cancer. Previous work has emphasized the context-dependent roles of the complement system in tumor initiation, inflammation, and immune escape, with most discussions centered on soluble complement effectors or broad pathway activation [31, 56]. By contrast, our data highlight CR1 as a gene with both protein-level and transcript-level relevance to HCC susceptibility and further suggest that part of this effect may be transmitted through specific circulating metabolites. This observation is important because it connects complement regulation to immunometabolic remodeling, a dimension that is increasingly recognized as critical for TAM function but has rarely incorporated into causal-inference frameworks in HCC [57].

The tissue-level and single-cell findings further extend the literature on TAM heterogeneity in HCC. Previous studies have shown that TAM abundance, M2 polarization, and PD-L1 expression are associated with impaired antitumor immunity and adverse outcomes in liver cancer [10, 58, 59]. Our results refine this concept by pinpointing CR1^+^ TAMs as a spatially restricted macrophage population enriched in advanced tumors, closely co-localized with CD206, and inversely associated with CD8^+^ T-cell infiltration. This spatially resolved view suggests that CR1 is not simply a diffuse inflammatory signal but may define discrete immunosuppressive niches within the HCC ecosystem, thereby helping to explain how local macrophage programs are translated into clinically relevant immune exclusion.

The in vitro experiments provide functional support for this model. CR1 overexpression promoted an M2-like phenotype characterized by increased IL-10, reduced TNF-a, enhanced phagocytosis, and higher PD-L1 expression, whereas CR1 knockdown shifted macrophages in the opposite direction. Moreover, CR1-high macrophages directly restrained CD8^+^ T-cell proliferation and attenuated IFN-gamma and granzyme B production in co-culture, which is consistent with previous evidence that macrophage checkpoint signaling and dysfunctional myeloid-T-cell crosstalk are central barriers to effective immunotherapy in HCC [8, 11, 60]. Taken together, these data suggest that CR1 may have translational value not only as a prognostic biomarker but also as a therapeutic entry point for macrophage reprogramming, particularly in combination with anti-PD-1/PD-L1-based strategies.

Another notable aspect of this work is the exploratory integration of the intratumoral microbiome with the CR1-immune axis. Although the microbiome analysis remains associative, the observed coupling between selected bacterial genera, immunoregulatory genes, and CTL-related programs raises the possibility that tumor-resident microbes participate in sustaining CR1-linked immunosuppressive macrophage states [61–63]. This observation broadens the conceptual scope of the study and underscores one of its key strengths: the use of orthogonal datasets and validation layers to interrogate CR1 biology from multiple angles rather than relying on a single omics modality or a single experimental system.

Several limitations should nevertheless be acknowledged. First, the MR analyses were derived primarily from datasets of European ancestry, and external validation in ethnically diverse cohorts remains necessary. Second, although THP-1-derived macrophages provided a tractable perturbation model, they cannot fully recapitulate the complexity of primary human TAMs in vivo. Third, the downstream signaling circuitry linking CR1 to macrophage polarization was not dissected in this study, and the microbiome findings remain hypothesis-generating rather than causal. Future work should therefore focus on validating CR1 in independent clinical cohorts, defining the signaling pathways that couple CR1 to M2-like reprogramming, and testing whether pharmacologic or genetic inhibition of CR1 can restore antitumor immunity and improve responsiveness to immune checkpoint blockade in preclinical HCC models.

## Conclusion

In summary, this study supports CR1 as a biologically meaningful and clinically relevant regulator of the HCC immune microenvironment. By integrating causal genetic analysis, multi-omics mapping, clinical validation, and functional perturbation, we show that CR1 is preferentially enriched in TAMs, associated with M2-like polarization and CD8^+^ T-cell dysfunction, and linked to aggressive tumor behavior. These findings provide new mechanistic insight into complement-associated immune escape in HCC and offer a rationale for developing CR1 as a biomarker for immune stratification and as a potential target for macrophage-centered combination immunotherapy. Future translational studies should determine whether CR1-directed intervention can synergize with checkpoint blockade and whether CR1 expression can help identify patients most likely to benefit from such strategies.

## Electronic supplementary material

Below is the link to the electronic supplementary material.

Supplementary Material 1

Supplementary Material 2

Supplementary Material 3

Supplementary Material 4

Supplementary Material 5

Supplementary Material 6

Supplementary Material 7

Supplementary Material 8

Supplementary Material 9

## Data Availability

The publicly available datasets analyzed in this study are available from the following repositories: FinnGen (https://www.finngen.fi/en), eQTLGen (https://www.eqtlgen.org), The Cancer Genome Atlas (TCGA; https://portal.gdc.cancer.gov/), and the Gene Expression Omnibus (GEO; https://www.ncbi.nlm.nih.gov/geo/) under accession numbers GSE149614 and GSE245908. Metabolomics GWAS data were obtained from the Canadian Longitudinal Study on Aging. Drug-response data were retrieved from the Genomics of Drug Sensitivity in Cancer (GDSC) database (https://www.cancerrxgene.org/). Protein structures were obtained from the AlphaFold database (https://alphafold.com/), and compound structures were retrieved from PubChem (https://pubchem.ncbi.nlm.nih.gov/). The clinical tissue samples and experimental data generated in this study are not publicly available due to ethical and privacy restrictions but are available from the corresponding author upon reasonable request and with permission from the Ethics Committee of Shandong Provincial Hospital Affiliated to Shandong First Medical University.

## Abbreviations

ANOVA: one-way analysis of variance
cDNA: complementary DNA
CFSE: carboxyfluorescein succinimidyl ester
CR1: complement receptor 1
CTL: cytotoxic T lymphocyte
eQTLs: expression quantitative trait loci
FDR: false discovery rate
FPKM: fragments per kilobase of transcript per million mapped reads
GDSC: Genomics of Drug Sensitivity in Cancer
GEO: Gene Expression Omnibus
GWAS: genome-wide association study
HCC: hepatocellular carcinoma
IC50: half-maximal inhibitory concentration
IF: immunofluorescence
IFN-γ: interferon-gamma
IHC: immunohistochemistry
IL-4: interleukin-4
IL-10: interleukin-10
IL-13: interleukin-13
IVs: instrumental variables
IVW: inverse-variance weighted
LD: linkage disequilibrium
MR: Mendelian randomization
MR-PRESSO: Mendelian Randomization Pleiotropy RESidual Sum and Outlier
PBMCs: peripheral blood mononuclear cells
PCA: principal component analysis
PD-L1: programmed death-ligand 1
pQTLs: protein quantitative trait loci
qRT-PCR: quantitative real-time polymerase chain reaction
RCTD: robust cell type decomposition
scRNA-seq: single-cell RNA sequencing
SEM: standard error of the mean
SNP: single-nucleotide polymorphism
ST: spatial transcriptomics
TAMs: tumor-associated macrophages
TCGA: The Cancer Genome Atlas
TGF-β: transforming growth factor-beta
TME: tumor microenvironment
TNF-α: tumor necrosis factor-alpha
Tregs: regulatory T cells
UMAP: Uniform Manifold Approximation and Projection
UMI: unique molecular identifier.
