# Supplementary figures and images for: CR1(+) tumor-associated macrophages orchestrate an immunosuppressive niche in hepatocellular carcinoma: a genetic and multi-omics dissection
Source: J Transl Med. 2026 May 25;24:955. doi: 10.1186/s12967-026-08301-z (PMC13397638; doi:10.1186/s12967-026-08301-z)

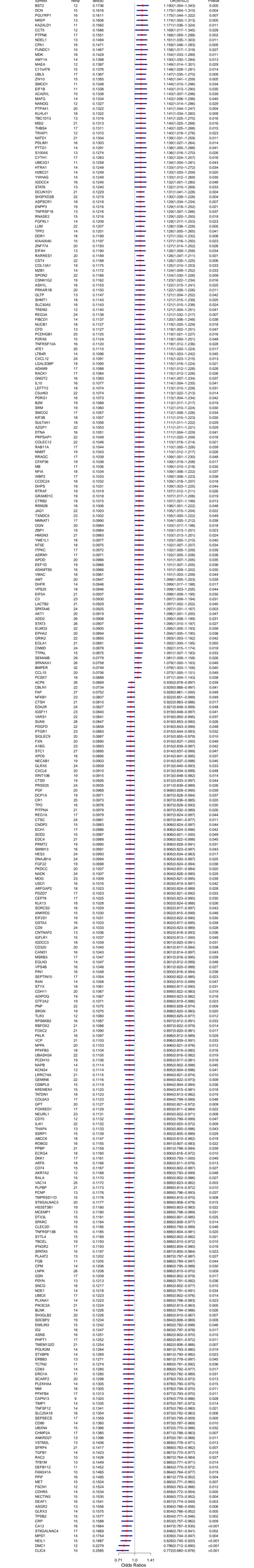

Supplement: Supplementary file 1 — Supplementary Material 1 [file 12967_2026_8301_MOESM1_ESM.pdf]

**A**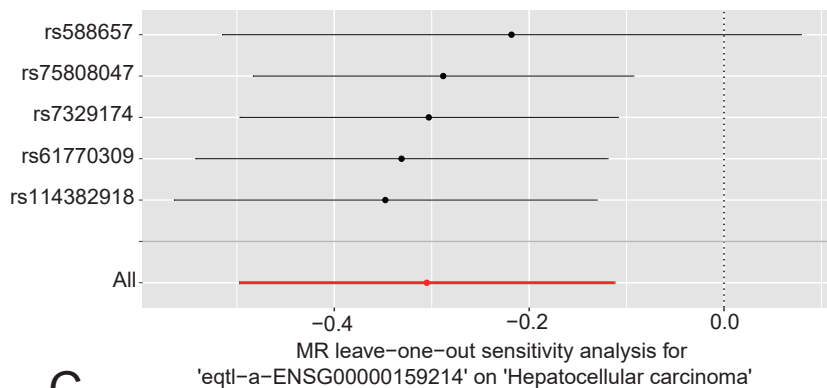**B**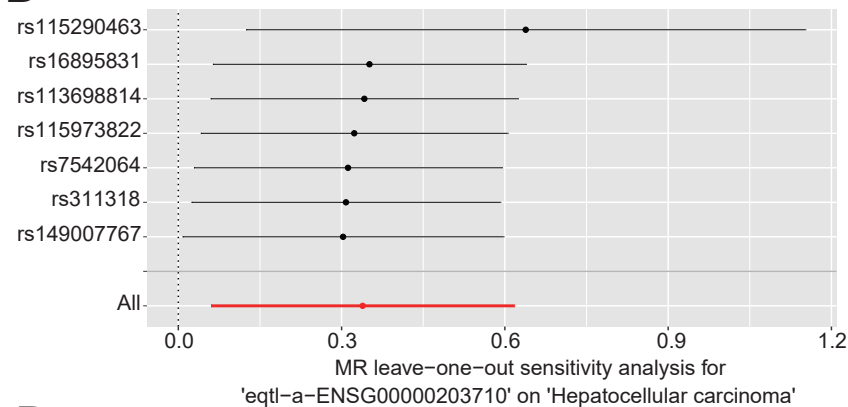**C**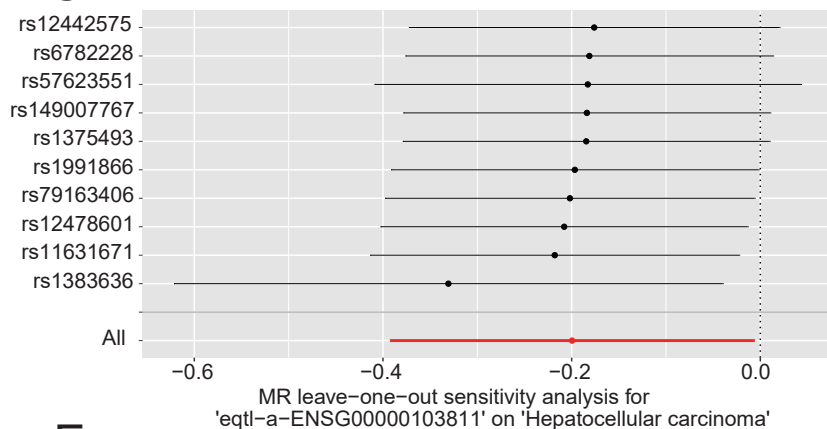**D**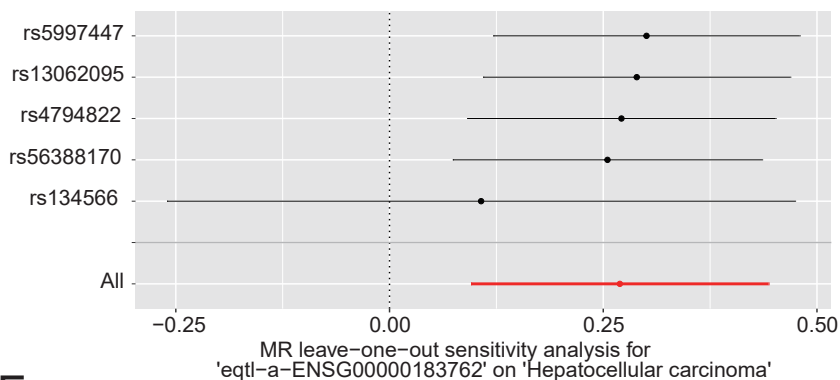**E**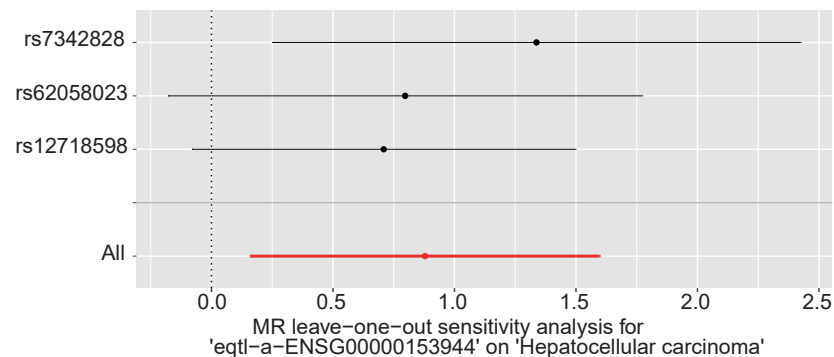**F**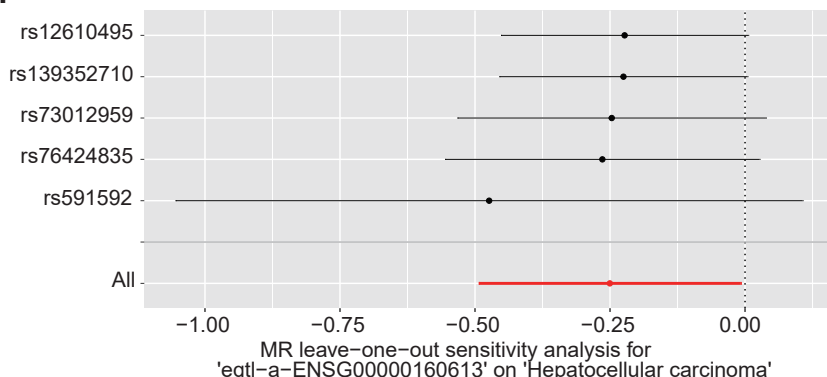**G**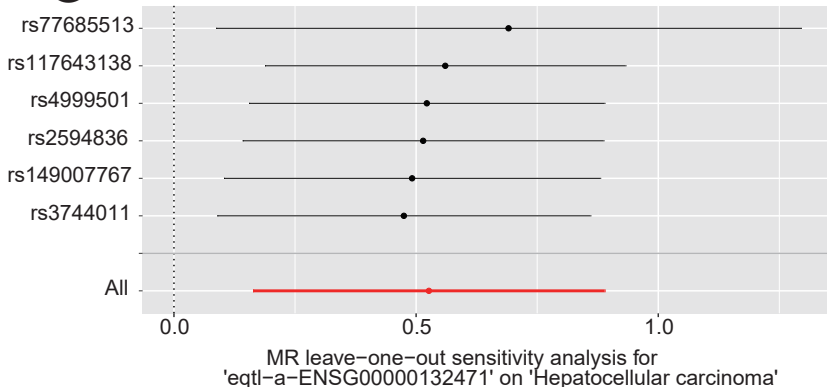**H**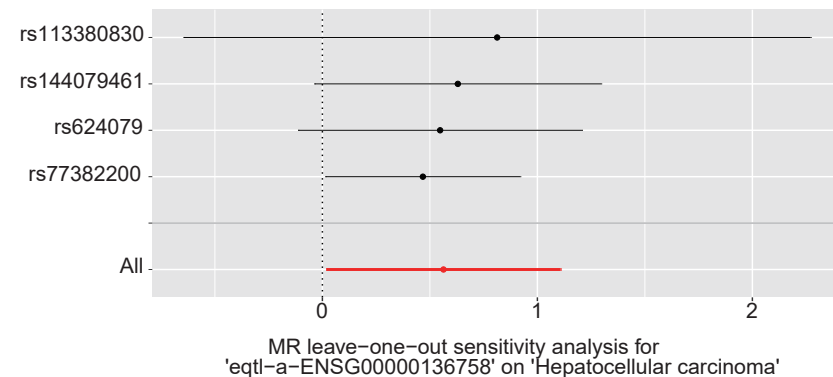

Supplement: Supplementary file 2 — Supplementary Material 2 [file 12967_2026_8301_MOESM2_ESM.pdf]

**A**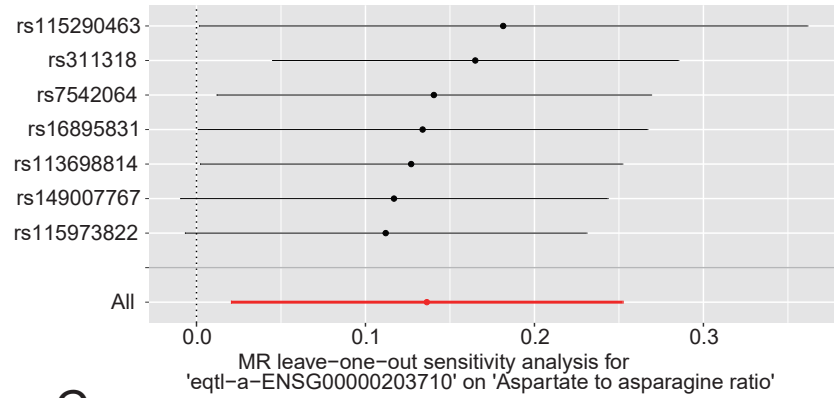**B**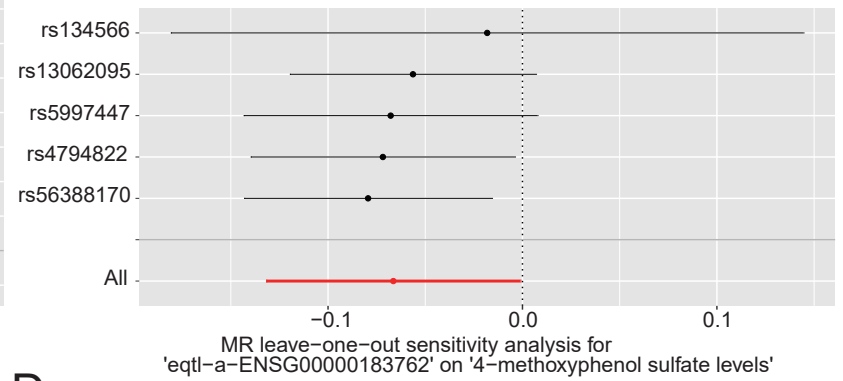**C**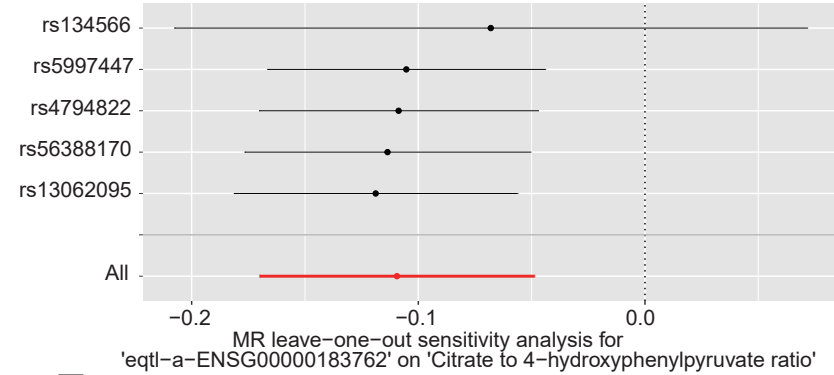**D**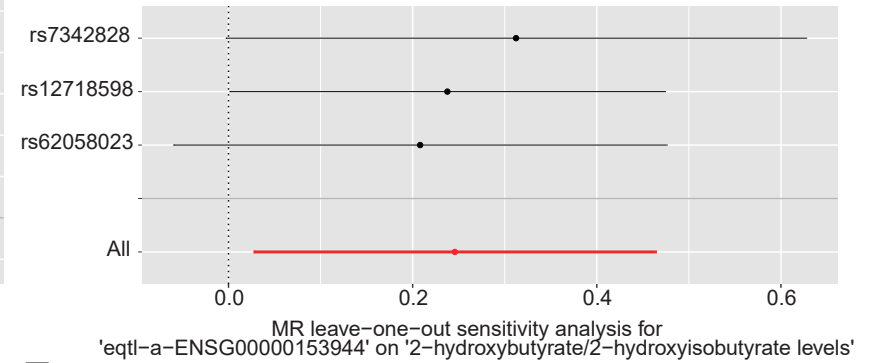**E**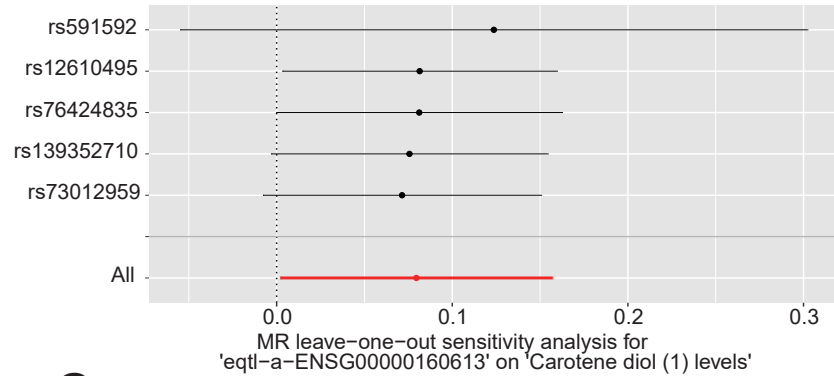**F**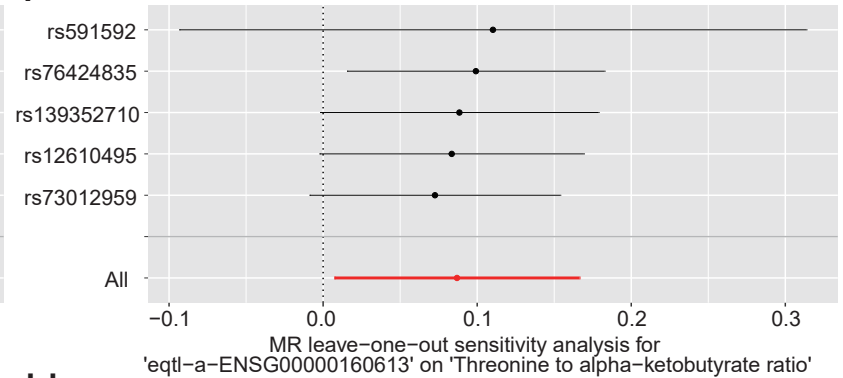**G**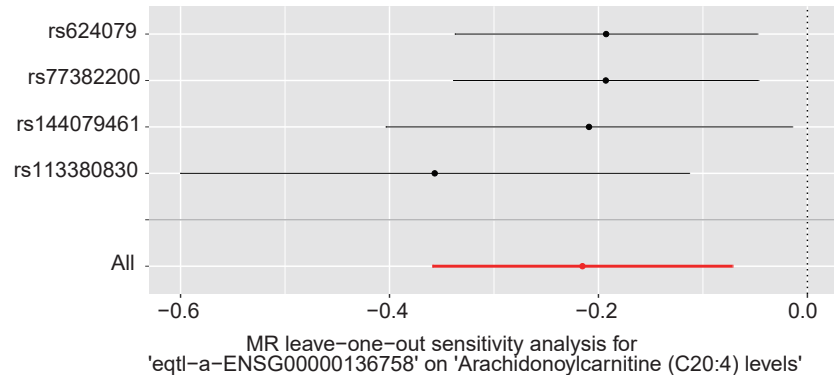**H**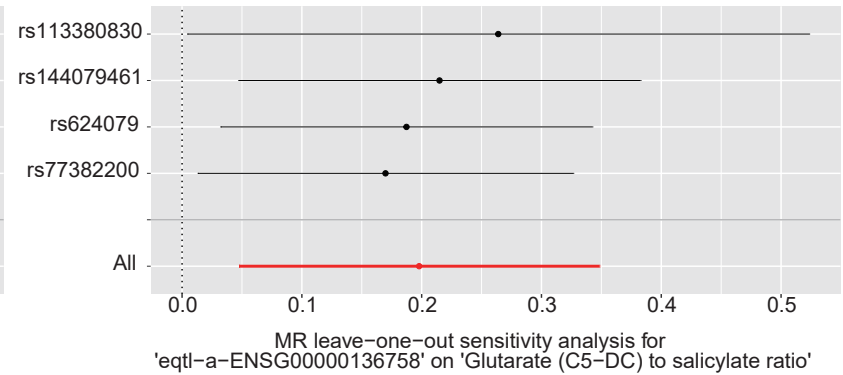

Supplement: Supplementary file 4 — Supplementary Material 4 [file 12967_2026_8301_MOESM4_ESM.pdf]

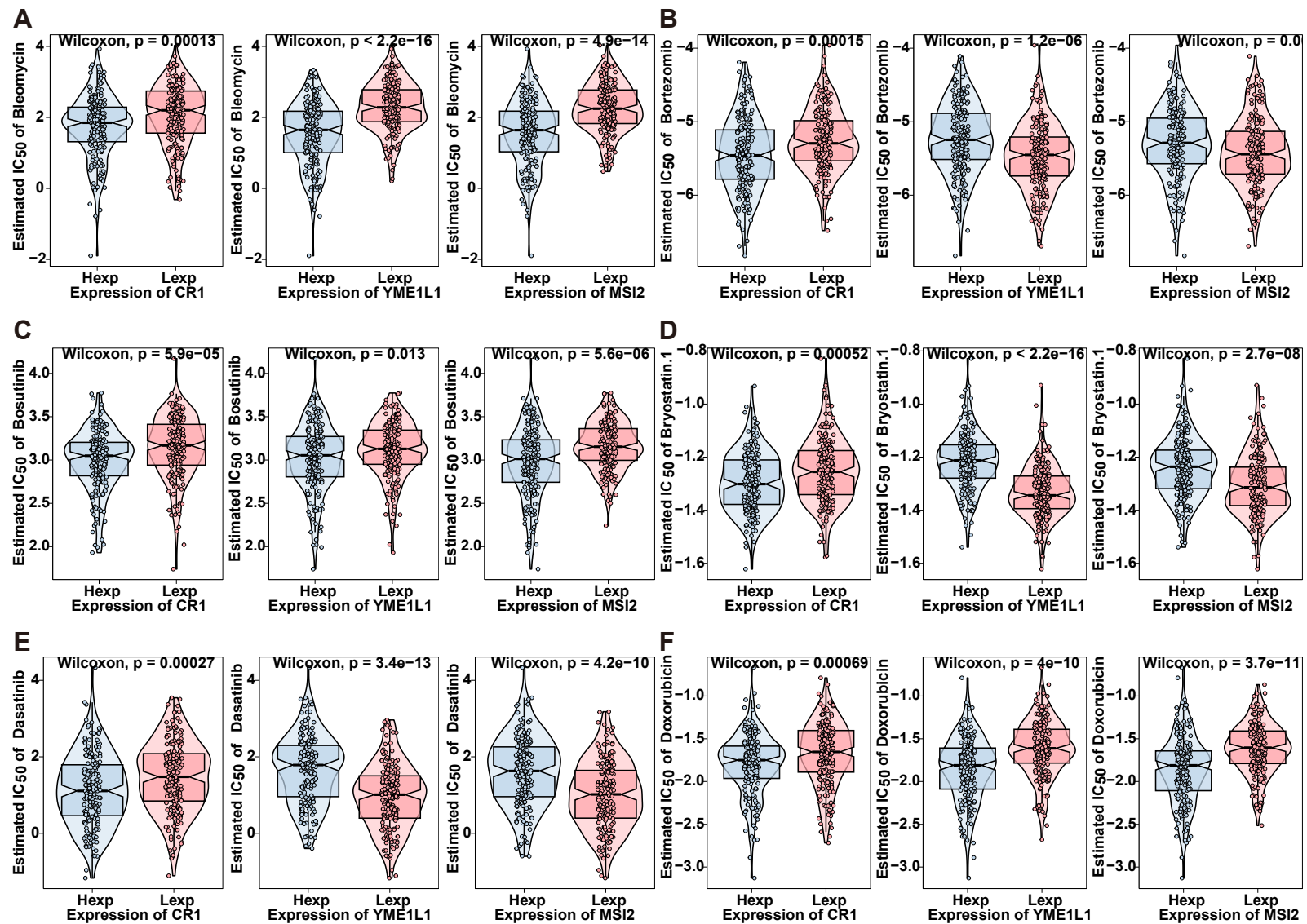

Supplement: Supplementary file 5 — Supplementary Material 5 [file 12967_2026_8301_MOESM5_ESM.pdf]

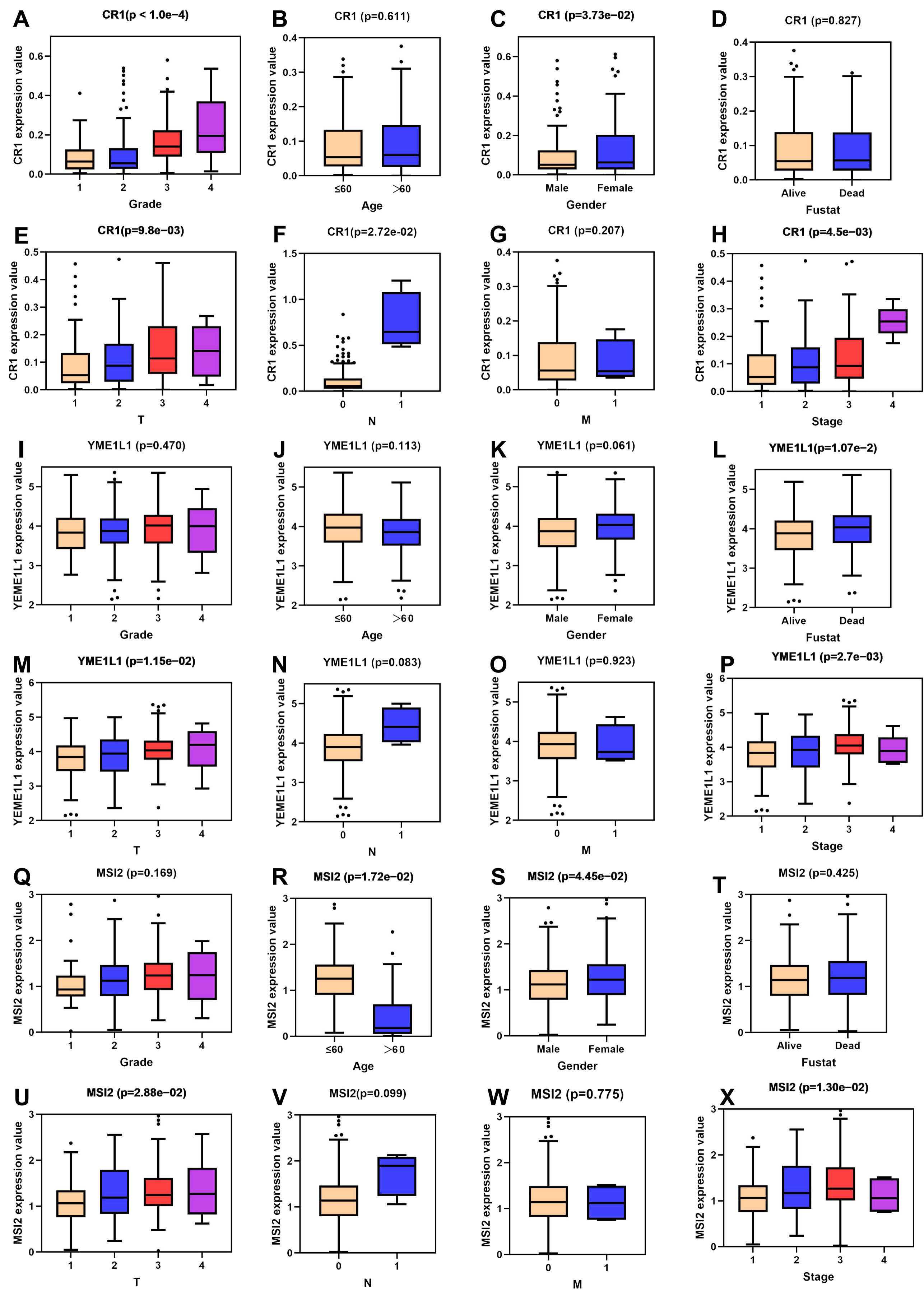

Supplement: Supplementary file 6 — Supplementary Material 6 [file 12967_2026_8301_MOESM6_ESM.pdf]

**A**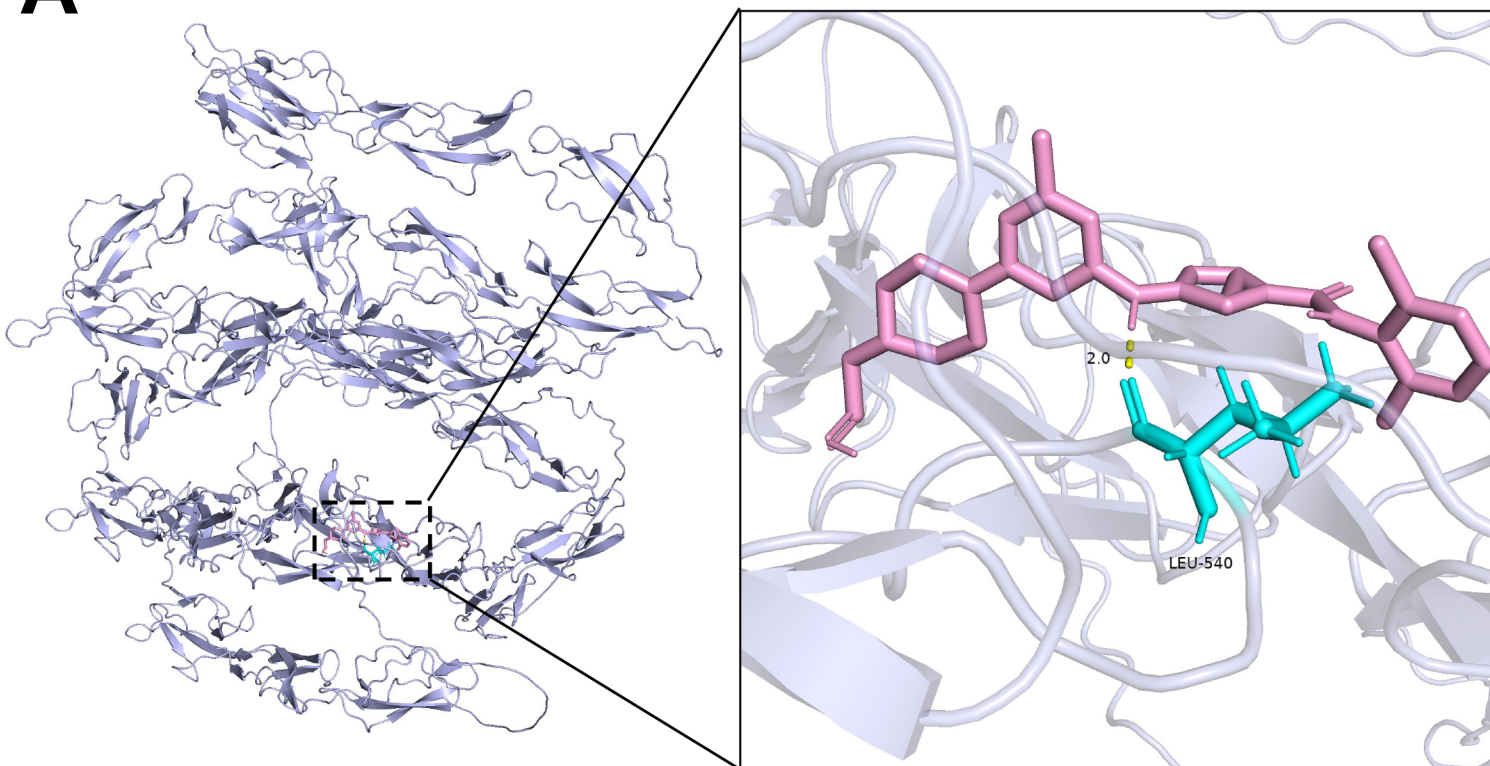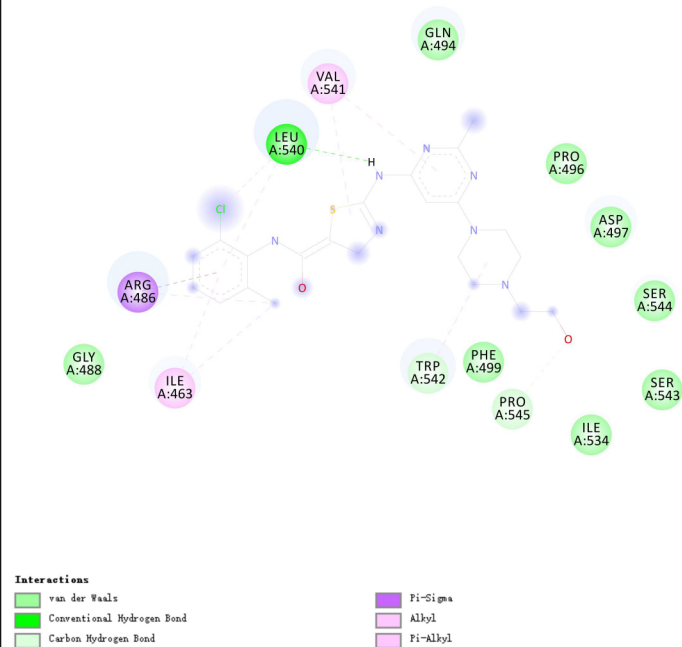**CR1-Dasatinib****B**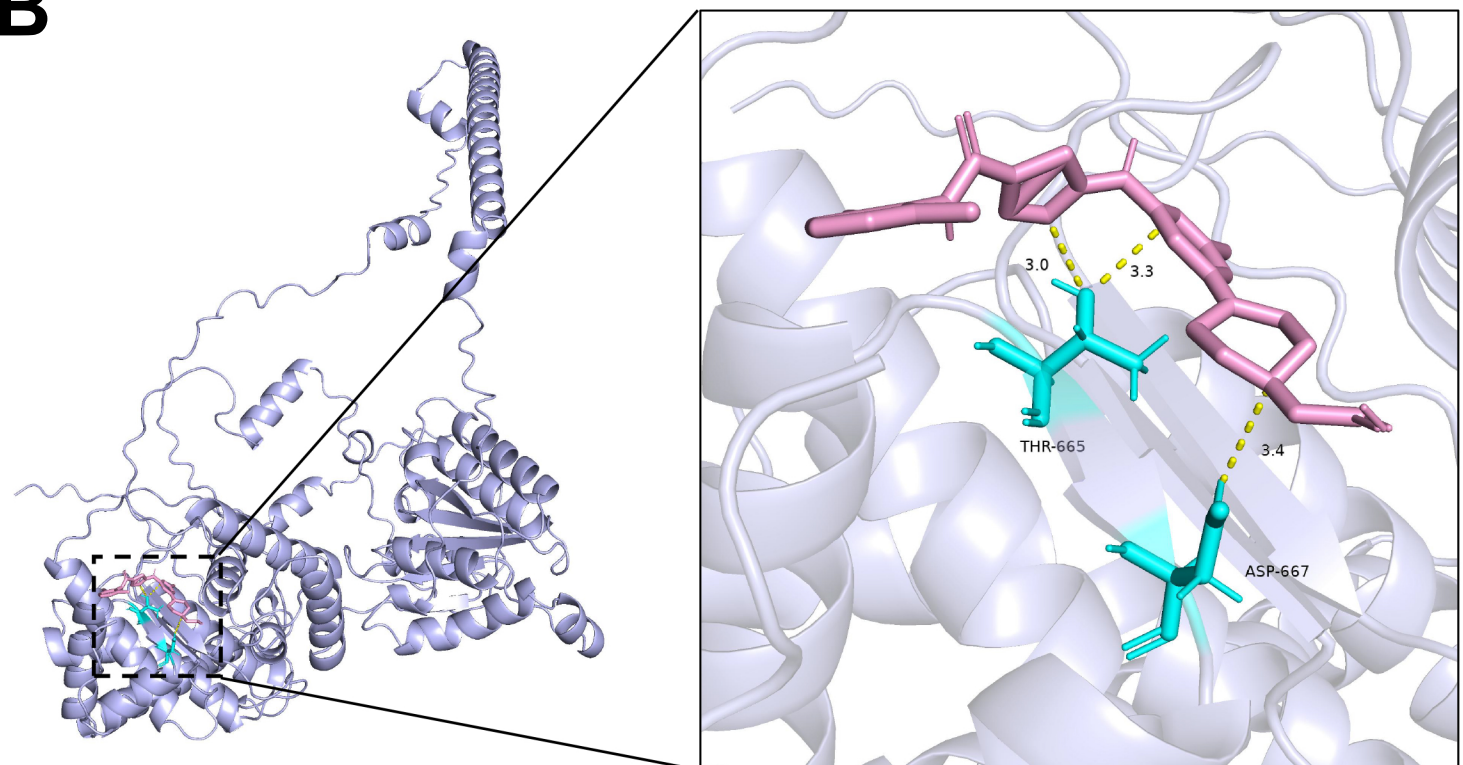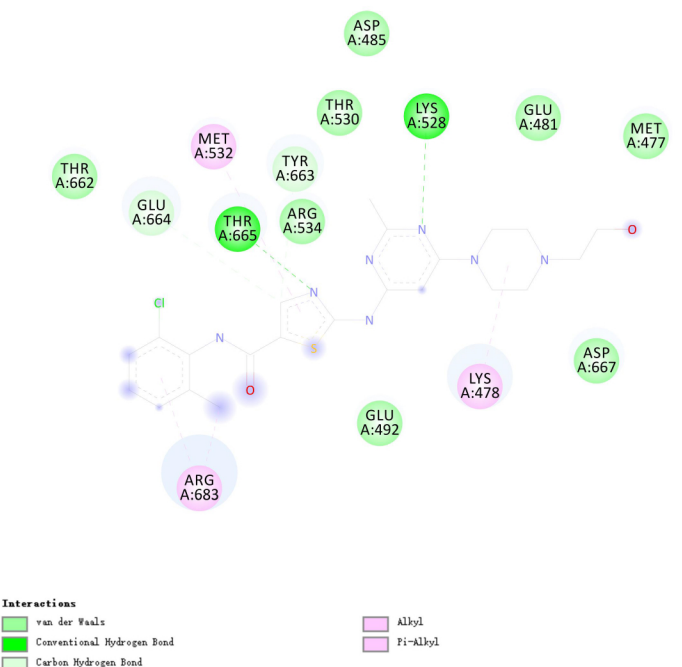**YME1L1-Dasatinib****C**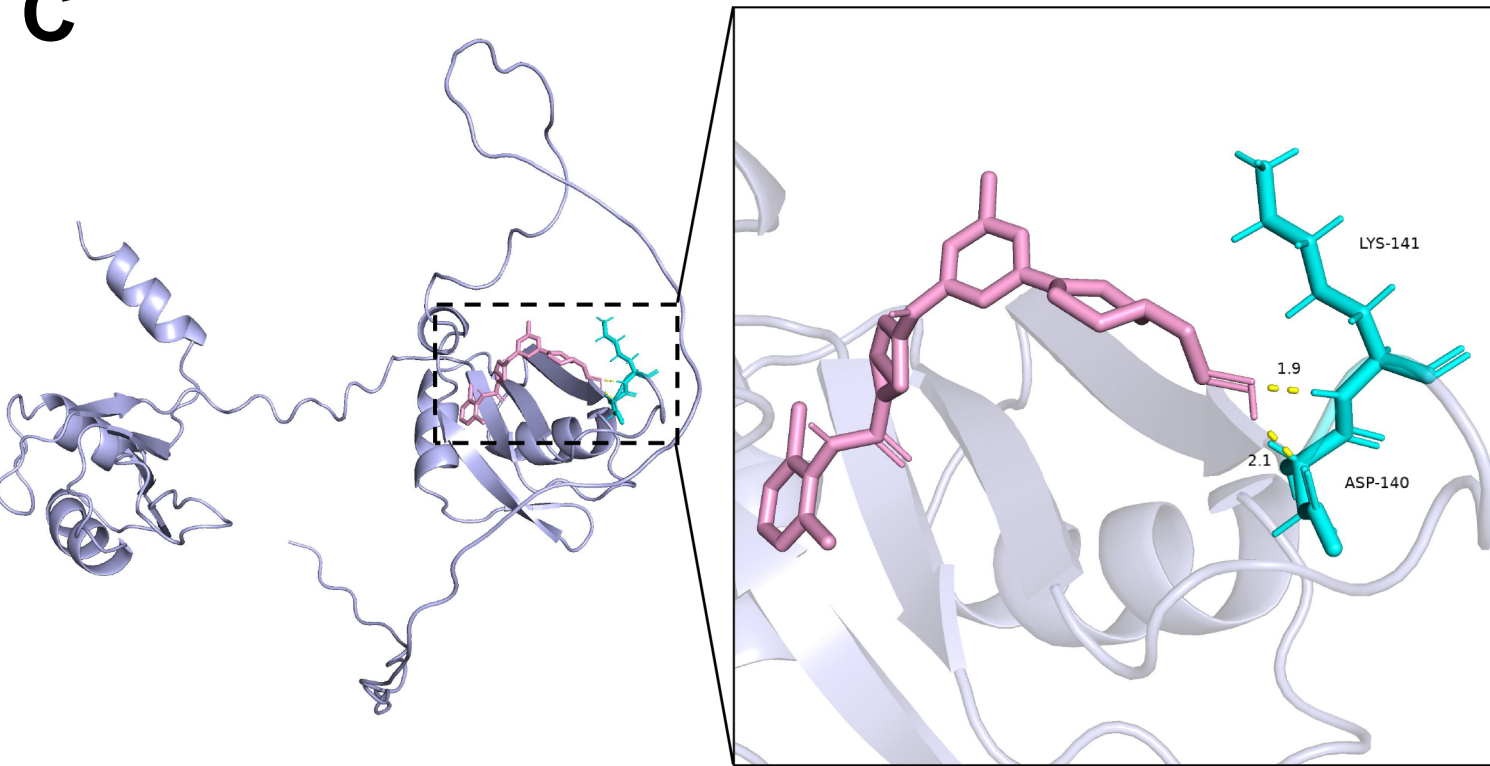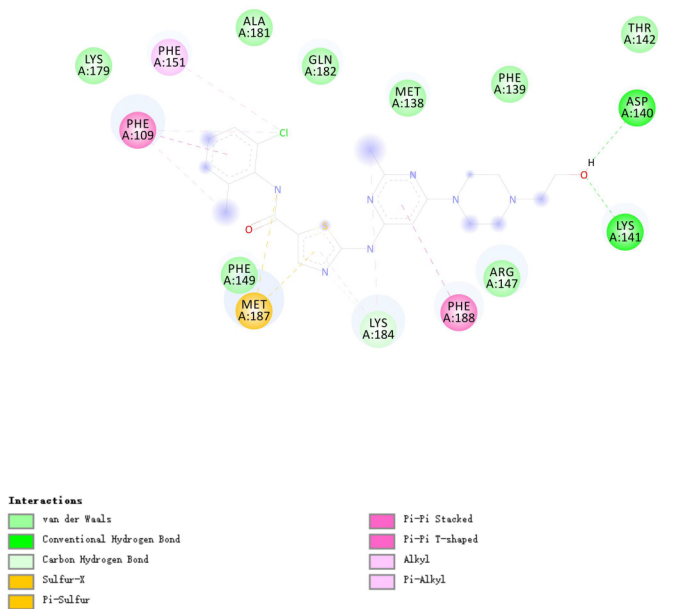**MSI2-Dasatinib**

Supplement: Supplementary file 7 — Supplementary Material 7 [file 12967_2026_8301_MOESM7_ESM.pdf]
