# Supplementary material for: CR1(+) tumor-associated macrophages orchestrate an immunosuppressive niche in hepatocellular carcinoma: a genetic and multi-omics dissection
Source: J Transl Med. 2026 May 25;24:955. doi: 10.1186/s12967-026-08301-z (PMC13397638; doi:10.1186/s12967-026-08301-z)

Metabolites	Nsnp	abs(B)	OR(95%CI)	Pvalue
4−methoxyphenol sulfate levels	20	0.4564	1.578(1.202−2.072)	0.001
2−hydroxybutyrate/2−hydroxyisobutyrate levels	17	0.4238	1.528(1.077−2.168)	0.018
Acetoacetate levels	15	0.4092	1.506(1.143−1.983)	0.004
Methionine to phosphate ratio	16	0.3962	1.486(1.070−2.065)	0.018
X−22776 levels	12	0.3768	1.458(1.006−2.111)	0.046
Gamma−tocopherol/beta−tocopherol levels	24	0.3738	1.453(1.110−1.903)	0.007
X−23636 levels	17	0.3493	1.418(1.039−1.936)	0.028
X−25519 levels	19	0.3378	1.402(1.044−1.883)	0.025
Urate levels	29	0.3178	1.374(1.111−1.699)	0.003
Citrate to 4−hydroxyphenylpyruvate ratio	20	0.3173	1.373(1.056−1.786)	0.018
Glutarate (C5−DC) to salicylate ratio	18	0.3138	1.369(1.032−1.815)	0.029
1−palmitoyl−2−palmitoleoyl−gpc (16:0/16:1) levels	23	0.3053	1.357(1.064−1.730)	0.014
Phenylalanine to phosphate ratio	23	0.2983	1.348(1.053−1.724)	0.018
Beta−hydroxyisovalerate levels	28	0.2861	1.331(1.055−1.680)	0.016
3−methoxycatechol sulfate (1) levels	22	0.2815	1.325(1.063−1.652)	0.012
Aspartate to asparagine ratio	19	0.2788	1.322(1.044−1.673)	0.021
Gentisate levels	28	0.2735	1.315(1.037−1.667)	0.024
1−(1−enyl−palmitoyl)−GPC (p−16:0) levels	26	0.2734	1.314(1.047−1.650)	0.019
3−hydroxy−2−methylpyridine sulfate levels	20	0.2728	1.314(1.014−1.702)	0.039
N−formylphenylalanine levels	32	0.2655	1.304(1.047−1.625)	0.018
Glucuronide of C12H22O4 (1) levels	18	0.2652	1.304(1.043−1.630)	0.020
Arachidonate (20:4n6) to paraxanthine ratio	22	0.2608	1.298(1.046−1.611)	0.018
X−12100 levels	19	0.2561	1.292(1.001−1.667)	0.049
3,7−dimethylurate levels	32	0.2525	1.287(1.066−1.555)	0.009
3−hydroxy−2−ethylpropionate levels	29	0.2474	1.281(1.036−1.584)	0.022
Alpha−hydroxyisovalerate levels	22	0.2379	1.269(1.035−1.555)	0.022
Arachidonate (20:4n6) to caffeine ratio	22	0.2341	1.264(1.007−1.586)	0.043
Cystathionine levels	35	0.2271	1.255(1.022−1.542)	0.031
Beta−hydroxyisovaleroylcarnitine levels	36	0.2181	1.244(1.005−1.539)	0.045
Cys−gly, oxidized levels	24	0.2171	1.242(1.016−1.519)	0.034
2−hydroxyphenylacetate levels	27	0.2129	1.237(1.010−1.515)	0.040
Hypotaurine levels	29	0.2115	1.236(1.006−1.518)	0.044
Glucose−to−mannose ratio	26	0.1997	1.221(1.006−1.482)	0.044
Arachidonoylcarnitine (C20:4) levels	35	0.1491	1.161(1.006−1.340)	0.041
X−25265 levels	28	0.1269	1.135(1.018−1.266)	0.023
Bilirubin degradation product, C17H20N2O5 (2) levels	24	0.1316	0.877(0.774−0.993)	0.039
Decadienedioic acid (C10:2−DC) levels	23	0.1376	0.871(0.761−0.998)	0.047
1−palmitoyl−2−linoleoyl−GPE (16:0/18:2) levels	24	0.1559	0.856(0.750−0.977)	0.021
1−oleoyl−2−linoleoyl−GPE (18:1/18:2) levels	32	0.1587	0.853(0.733−0.994)	0.041
Bilirubin degradation product, C17H20N2O5 (1) levels	24	0.1846	0.831(0.734−0.942)	0.004
X−18922 levels	29	0.2164	0.805(0.668−0.972)	0.024
Gamma−glutamyltyrosine levels	28	0.2182	0.804(0.652−0.991)	0.041
X−23739 levels	27	0.2185	0.804(0.654−0.987)	0.037
Sphingomyelin (d18:1/20:2, d18:2/20:1, d16:1/22:2) levels	26	0.2341	0.791(0.646−0.969)	0.024
Sphingomyelin (d18:1/18:1, d18:2/18:0) levels	34	0.2356	0.790(0.640−0.975)	0.028
Phosphoethanolamine levels	24	0.2376	0.789(0.647−0.962)	0.019
Picolinate levels	23	0.2403	0.786(0.628−0.984)	0.036
1−stearoyl−GPG (18:0) levels	28	0.2409	0.786(0.640−0.966)	0.022
1,2−dilinoleoyl−GPC (18:2/18:2) levels	17	0.2437	0.784(0.627−0.979)	0.032
Glutamine levels	26	0.2480	0.780(0.616−0.988)	0.039
Alpha−ketoglutarate to kynurenine ratio	26	0.2552	0.775(0.631−0.951)	0.015
Carotene diol (1) levels	33	0.2582	0.772(0.636−0.938)	0.009
Adenosine 5'−monophosphate (AMP) to glycine ratio	23	0.2611	0.770(0.616−0.962)	0.022
1,2−dilinoleoyl−GPE (18:2/18:2) levels	28	0.2622	0.769(0.636−0.930)	0.007
Spermidine to carnitine ratio	19	0.2648	0.767(0.595−0.990)	0.042
Eicosanedioate (C20−DC) levels	14	0.2949	0.745(0.577−0.961)	0.024
Aspartate to citrulline ratio	19	0.3027	0.739(0.551−0.990)	0.043
Threonine to alpha−ketobutyrate ratio	20	0.3239	0.723(0.556−0.941)	0.016
Valine levels	22	0.3384	0.713(0.523−0.971)	0.032
2'−deoxyuridine levels	24	0.3463	0.707(0.558−0.896)	0.004
1−linoleoylglycerol (18:2) levels	18	0.3532	0.702(0.507−0.973)	0.034
3−methyl−2−oxovalerate to 4−methyl−2−oxopentanoate ratio	22	0.3781	0.685(0.493−0.951)	0.024
Serine to alpha−ketobutyrate ratio	17	0.4204	0.657(0.496−0.870)	0.003
Salicylate to citrate ratio	22	0.4253	0.654(0.511−0.836)	<0.001

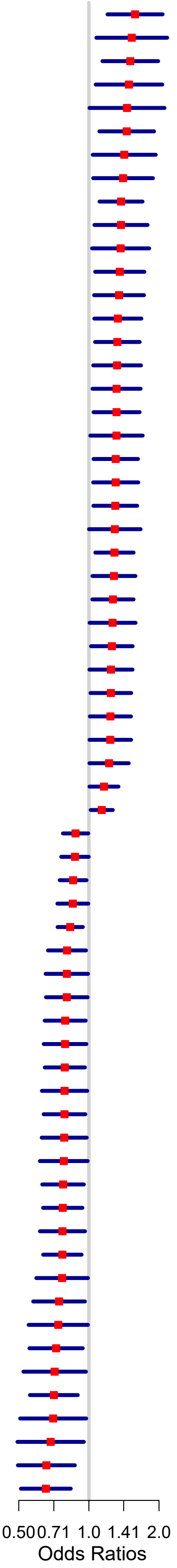

Supplement: Supplementary file 3 — Supplementary Material 3 [file 12967_2026_8301_MOESM3_ESM.pdf]
