# Supplementary material for: CR1(+) tumor-associated macrophages orchestrate an immunosuppressive niche in hepatocellular carcinoma: a genetic and multi-omics dissection
Source: J Transl Med. 2026 May 25;24:955. doi: 10.1186/s12967-026-08301-z (PMC13397638; doi:10.1186/s12967-026-08301-z)
Supplement: Supplementary file 8 — Supplementary Material 8 [file 12967_2026_8301_MOESM8_ESM.pdf]

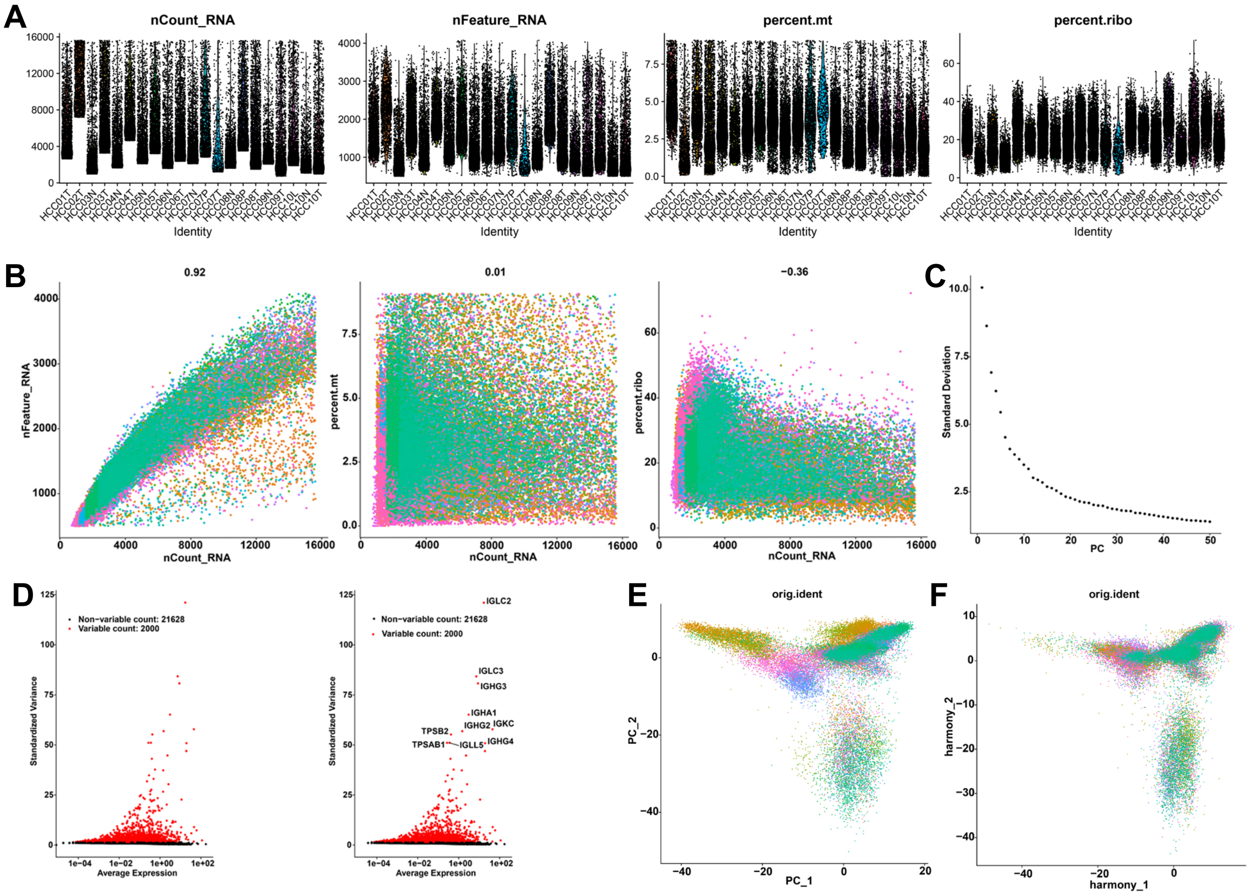
A
nCount_RNA
nFeature_RNA
percent.mt
percent.ribo
Identity
HCC01T HCC02T HCC03N HCC03T HCC04N HCC04T HCC05N HCC05T HCC06N HCC06T HCC07N HCC07P HCC07T HCC08N HCC08P HCC08T HCC09N HCC09T HCC10L HCC10N HCC10T
B
0.92
0.01
−0.36
nFeature_RNA
percent.mt
percent.ribo
nCount_RNA
C
Standard Deviation
PC
D
Non−variable count: 21628
Variable count: 2000
Standardized Variance
Average Expression
IGLC2
IGLC3
IGHG3
IGHA1
IGHG2
IGKC
TPSB2
TPSAB1
IGLL5
IGHG4
E
orig.ident
PC_1
PC_2
F
orig.ident
harmony_1
harmony_2
